# Certified Elimination of Source Candidates Under Capacity, Transit-Time, and Deadline Constraints

**DOI:** 10.3390/e28091022

**Published:** 2026-09-14

**Authors:** Zimeng Wang, Chao Zhao, Chung Chan

**Affiliations:** Department of Computer Science, City University of Hong Kong, Hong Kong, China; zimenwang4-c@my.cityu.edu.hk (Z.W.); ao.ao@my.cityu.edu.hk (C.Z.)

**Keywords:** source identification, flows over time, network coding, minimum cut, minimum-cost circulation, transit time, candidate elimination

## Abstract

Source identification is constrained not only by network connectivity but also by whether a finite message can reach observed nodes before a deadline. We study deterministic source-candidate elimination in directed networks with arc capacities and transit times. A time-expanded construction gives an exact causal network-coding characterization in which a candidate is retained if and only if its temporal min-cut to every required recipient is at least the message size. Rejection is therefore conservative for any weaker compliant routing or replication protocol. We derive an equivalent minimum-cost circulation computation, monotone certificates under parameter uncertainty, and closed-form formulas for bidirected trees, including linear-time evaluation for uniform capacities and an $O(|V|\log^{2}|V|)$ centroid decomposition algorithm for heterogeneous capacities. Protocol-generated experiments show zero true-source eliminations and substantial refinement in routing regimes. Held-out calibration supports transfer to unseen networks. On benchmark representations of 40 real backbone topology families with 50 to 197 nodes, mean candidate retention falls from 99.3% under static screening to 25.3% under exact temporal screening, without true-source elimination. A focused RLNC diagnostic attributes weak refinement in coding-rich regimes to the static screen retaining all candidates and to broad temporal feasibility, while decode-before-forward restrictions create a substantially larger protocol gap.

## 1. Introduction

Source identification is commonly posed as an inference problem. Given an observed diffusion pattern, a probabilistic model, a learned predictor, or a graph-based centrality measure ranks candidate nodes according to how plausibly they could have initiated the spread. Such methods are useful when the objective is to identify the most likely source, but their conclusions depend on assumptions about the spreading law, observation process, or training distribution. A complementary question can be answered without assigning likelihoods. Given explicit transport constraints, can a candidate be ruled out because the observed delivery could not have originated from that node?

Our previous work introduced an information-flow approach to this question [1]. Rather than estimating a source probability, it characterizes candidates that can support a required multicast rate to the observed nodes. This formulation connects source identification with minimum cuts, network coding, and scalable recipient-wise feasibility computations. However, a static rate condition does not describe the delivery of a finite message before a deadline. A low-capacity path may eventually transmit any finite payload, whereas a high-capacity path is unusable when its transit time already exceeds the available horizon. Static capacity and temporal feasibility must therefore be distinguished.

This article develops a finite-message, finite-time counterpart of the earlier information-flow formulation. A message of *K* symbols is completely available at the source at time zero. Each arc has a per-slot capacity and a positive transit time, and a set of observed nodes is known to possess the complete message by a common deadline *H*. The states of all other nodes are unspecified. They may possess the complete message, only part of it, or no part of it, and they may have forwarded symbols without being observed. The candidate-source domain is specified independently of the observed set. For a candidate *s*, the required recipients are the observed nodes other than *s* itself.

The objective is deterministic candidate elimination. A candidate is retained when causal network coding can deliver the message from that candidate to every required recipient by the deadline. Otherwise, it is rejected. Network coding is not assumed to describe ordinary diffusion behavior. It is used as an upper bound on transport capability. Consequently, rejection remains valid for any less powerful routing, copying, or replication process that obeys the same arc capacities and transit times. Retention has the weaker interpretation that the candidate has not been disproved by the stated transport model; it does not imply that the candidate is likely or that a particular uncoded protocol can realize the delivery.

The exact characterization follows from applying the single-source multicast theorem to a time-expanded network. A candidate is retained if and only if the temporal minimum cut from that candidate to every required recipient is at least the message size. Pairwise temporal values can also be computed on the original network through the classical Ford–Fulkerson reduction from maximum flow over time to minimum-cost circulation [2,3,4,5]. This combination yields an exact coded retained set without requiring explicit construction of the full time-expanded graph for every candidate–recipient pair.

Several further results make the characterization usable. Shortest-transit and static-cut conditions provide conservative preliminary screens before exact computation. Monotonicity with respect to the message size, deadline, capacities, and transit times yields endpoint certificates under bounded parameter uncertainty. On bidirected trees, the pairwise temporal value reduces to a closed-form expression involving the total path transit time and the minimum path capacity. Under uniform capacities, this expression gives an intersection of recipient-centered metric balls and supports linear-time evaluation of all candidates. Under heterogeneous capacities, a centroid decomposition algorithm evaluates all candidates in $O(|V|\log^{2}|V|)$ time.

The experimental program follows the same progression as the theory. Controlled protocol observations first test safety and selectivity. Staged screening, recipient ordering, and tree algorithms then test computational efficiency. Held-out trees test transfer beyond calibrated instances. GEANT2009 and 40 backbone topology families test behavior on real network topologies. An independent normalization audit tests whether transformed transport attributes preserve shortest-path geometry. An RLNC diagnostic finally characterizes when the coded retained set is narrow and when it remains broad under actual protocols.

## 2. Related Work

### 2.1. Probabilistic and Learning-Based Source Identification

Classical source-identification methods infer the origin of a diffusion under an assumed spreading process. For susceptible–infected diffusion on trees, rumor centrality provides a maximum-likelihood estimator and permits asymptotic analysis of its detection probability [6]. Sample-path methods extend source inference to susceptible–infected–recovered processes by identifying propagation histories that best explain the observed state [7]. Other probabilistic formulations jointly estimate the source and elapsed spreading time under continuous-time diffusion models [8].

Timing information may also be obtained from a subset of monitored nodes. Pinto et al. use infection timestamps recorded by sparse observers to localize the source [9]. Farajtabar et al. first infer a continuous-time diffusion network from historical cascades and then estimate the source of a partially observed cascade [10]. Partial snapshots without complete node-state information have also been studied under SI dynamics using most-likely infection paths and Jordan-center estimators [11]. Bayesian belief propagation and dynamic message passing provide related inference frameworks for SI/SIR dynamics with missing observations or unknown observation times [12,13]. In these models, transmission times are random variables associated with the spreading process and enter the source estimator through a likelihood or related statistical objective.

Learning-based methods avoid direct evaluation of a diffusion likelihood. Graph-convolutional models learn node representations from topology and simulated infection states, including settings with multiple sources [14]. Graph-transformer models use attention mechanisms to incorporate longer-range structural dependencies and temporal or infection-state features [15]. Their predictions depend on the propagation, observation, and graph distributions represented in the training data.

Several methods produce a set of plausible sources rather than a single estimate. Confidence-set procedures seek a node set containing the true source with a prescribed probability under a specified stochastic model [16]. Such sets provide statistical coverage, whereas the retained set studied here is defined by deterministic transport feasibility. The two types of sets therefore have different guarantees and interpretations.

### 2.2. Application-Oriented Source Localization

Source localization is motivated by operational problems in which an investigator must narrow a large candidate set from incomplete observations. Sparse-sensor epidemic localization and partially observed cascade reconstruction use network structure and timing evidence to infer an origin [9,10,13]. Source identification on social graphs is also addressed by learning-based models that use simulated or historical diffusion data to rank candidate nodes [14,15].

Application-specific studies show how the available localization evidence changes across domains. Shah and Zaman studied the source of a computer virus under an epidemic spreading model and constructed an estimator from infected network observations [17]. In production data center networks, NetBouncer localizes failed devices and links from actively probed paths and was deployed in Microsoft Azure data centers [18]. In power systems, graph-based disturbance localization has been studied using measurements on weighted networked dynamic models, and dissipating energy flow derived from phasor measurement unit data has been used to trace sustained oscillation sources in simulated and actual grid events [19,20].

These application settings are related by the goal of narrowing candidate locations from network observations, but their physical models are not interchangeable. Computer virus diffusion is stochastic, data center fault localization diagnoses failed components rather than a diffusion origin, and power grid oscillations follow electromechanical dynamics rather than packet transport. The most direct interpretation of the present capacities, transit times, message size, and deadline is therefore in communication and distributed systems, where throughput, propagation or serialization delay, payload size, and completion deadlines are measurable. In another domain, the corresponding transport quantities must be justified by its physical model before the feasibility certificate is applied.

The present framework serves a complementary operational role. It does not replace a probabilistic, forensic, or learned ranking. Instead, it supplies a transport-feasibility certificate that can remove candidates unable to deliver the required information under stated capacities, transit times, and deadlines. A retained node still requires external evidence for attribution, whereas a rejected node is inconsistent with the specified transport model.

### 2.3. Network Information Flow

Network information-flow theory studies communication when intermediate nodes may route or combine received symbols. For single-source common-message multicast, the achievable rate is determined by the minimum source–terminal cut [3]. Linear operations are sufficient to attain multicast capacity over an appropriate finite field [21]. Algebraic formulations make the dependence on local coding coefficients explicit [22], while random linear network coding provides a distributed multicast construction that succeeds with high probability over a sufficiently large field [23]. Network coding differs from independently routing one copy of the message to each recipient because coded transmissions may reuse network resources across several destinations.

These results provide a capacity-based description of communication that is stronger than ordinary reachability. They are therefore useful whenever feasibility depends on how much information must traverse a network rather than only on whether paths exist. Classical multicast theory is predominantly static, however, and characterizes sustainable rates without explicitly representing a finite delivery horizon. The incorporation of arc transit times and a common deadline instead places the problem within the literature on flows over time.

### 2.4. Flows over Time and Deadline-Constrained Communication

Flows over time extend static network flows by assigning a transit time to each arc. Ford and Fulkerson related maximum flow within a fixed horizon to a static minimum-cost flow construction [2]. The complementary quickest-flow problem minimizes the horizon required to send a prescribed amount [24]. Time-expanded networks provide a discrete representation in which node copies correspond to time layers and arcs represent transmission or storage. Subsequent research has studied maximum dynamic flows, quickest flows, and transshipments, as well as algorithms that avoid explicitly constructing an expansion whose size grows with the time horizon [4,5,25].

Quickest-flow and transshipment formulations generally assume that the source locations, supplies, and destinations are known. Their objective is to determine a transmission plan or the earliest feasible completion time. Source-candidate analysis instead requires repeated feasibility evaluation for different possible origins of the same observed delivery. In addition, common-message multicast differs from transshipment because all recipients require the same information rather than independent portions of a supply.

Deadline-constrained network coding has also been investigated as a forward resource-allocation problem. Time-unwrapped networks have been used to optimize multicast coding and scheduling under wireless interference and packet deadlines [26]. Other models consider successive messages with individual expiration times, overlapping coding generations, or continuously arriving perishable information [27,28]. These traffic models differ from the single finite message that is completely available at time zero and subject to one common completion deadline.

Robust flows over time consider uncertain transit times, disruptions, or other adverse network realizations and seek flows that maintain performance across uncertainty scenarios [29]. This is distinct from determining whether the classification of a candidate remains unchanged over a bounded range of input parameters. The former optimizes a transmission policy under uncertainty, whereas the latter studies the stability of a feasibility decision.

### 2.5. Tree-Based Source and Location Problems

Trees support exact analysis in many source-localization models because every pair of nodes is connected by a unique path. Under susceptible–infected diffusion, likelihood estimators on regular trees are closely related to rumor centers [6]. Sample-path and center-based methods use quantities such as infection eccentricity, Jordan centers, or distances to the observed infected region [7]. These objectives are derived from diffusion likelihoods or path geometry rather than from the delivery of a capacity-constrained finite message.

Dynamic sink-location and evacuation problems also combine tree paths, arc capacities, and transit times. Supplies distributed across the vertices are routed toward one or more selected sinks, and the objective is commonly to minimize evacuation time, transportation cost, or regret [30,31]. Although both settings involve temporal flow on trees, evacuation sends independent supplies inward to a sink, whereas multicast sends one common message outward toward several recipients. The two problems therefore have different flow-sharing structures.

Centroid decomposition is a general technique for all-node queries on trees [32]. Recursively removing a centroid produces a decomposition of logarithmic depth and allows path information to be aggregated across separated components. It is widely used for distance queries, nearest marked-node problems, and other objectives whose candidate–terminal contributions can be combined through a separating centroid. In capacity-constrained temporal transport, the relevant path information includes both an additive transit-time term and a bottleneck-capacity term, requiring summaries different from conventional distance-only queries.

## 3. Model and Exact Candidate Characterization

### 3.1. Network, Message, and Observation Model

We consider the transmission of a finite message over a directed network subject to arc-capacity, transit-time, and deadline constraints. Let G=(V,E) be a finite directed network. We refer to each directed link as an *arc*. For B⊆V, let

(1) $$\delta^{+}\left(B\right):=\left\{\left(u,v\right)\in E:u\in B,\;v\notin B\right\},\;\delta^{-}\left(B\right):=\left\{\left(u,v\right)\in E:u\notin B,\;v\in B\right\},$$

and write $\delta^{\pm }\left(v\right):=\delta^{\pm }\left(\left\{v\right\}\right)$ for a single node. The symbol $\delta^{\pm }$ is reserved throughout for directed arc boundaries. Each arc e∈E has an integer capacity $c_{e}>0$, measured in symbols per time slot, and an integer transit time $\tau_{e}\geq 1$, measured in slots. In each slot, at most $c_{e}$ new symbols may enter arc *e*. Symbols entering at time *t* arrive at the head of the arc at time $t+\tau_{e}$, and transmissions initiated in different slots may overlap in transit.

For distinct nodes s,a∈V, let $P_{sa}$ denote the set of directed *s*–*a* paths and define the shortest-transit distance

(2) $$d_{\tau }\left(s,a\right):=\min_{P\in P_{sa}}\sum_{e\in P}\tau_{e},$$

with $d_{\tau }\left(s,a\right)=+\infty$ when $P_{sa}=⌀$.

A single finite message contains K≥0 independent symbols over a finite field. The complete message is available at the source at time zero, and no additional source symbols are generated afterward. Let

(3) $$T_{H}:=\left\{0,1,\ldots ,H\right\}$$

be the discrete time horizon, where H≥0 is the deadline. Arrival at time *H* is considered on time.

An arc *e* may contribute to on-time delivery only from departure slots *t* satisfying $t+\tau_{e}\leq H$. Its usable departure set is $\left\{0,\ldots ,H-\tau_{e}\right\}$ when $H\geq \tau_{e}$ and is empty otherwise. Hence, its number of usable departures is

(4) $${\left[H-\tau_{e}+1\right]}_{+},\;{\left[x\right]}_{+}:=\min\left\{x,0\right\}.$$

**Figure 1:**
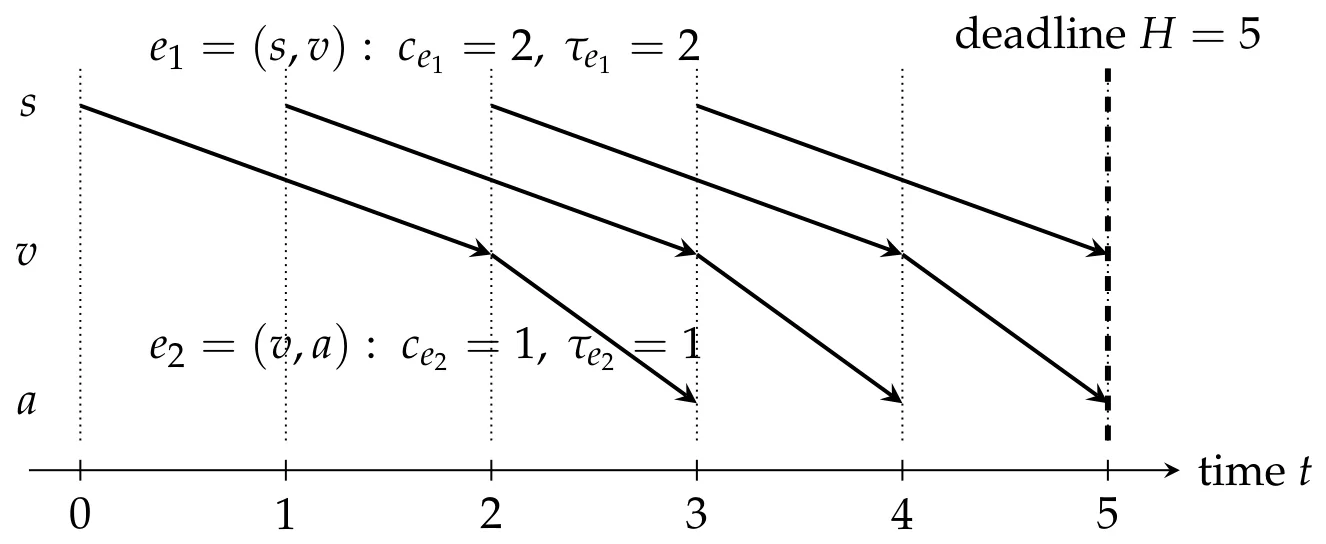
Time–space representation of transmissions along the path s→v→a. Each diagonal arrow represents one departure opportunity, not one individual symbol. An arrow associated with arc *e* may carry up to $c_{e}$ symbols, and its horizontal displacement equals the transit time $\tau_{e}$. The source may use (s,v) at times 0, 1, 2, and 3. Because source information first reaches *v* at time 2, the relay may forward that information through (v,a) at times 2, 3, and 4, with arrivals at *a* at times 3, 4, and 5, respectively.

Figure 1 illustrates how capacity, transit time, and causality interact along a two-arc path.

In Figure 1, arc $e_{1}=\left(s,v\right)$ has capacity $c_{e_{1}}=2$ and transit time $\tau_{e_{1}}=2$. Under deadline H=5, it has ${\left[5-2+1\right]}_{+}=4$ usable departure slots. Arc $e_{2}=\left(v,a\right)$ has capacity $c_{e_{2}}=1$ and transit time $\tau_{e_{2}}=1$, but source information first reaches *v* at time 2. Hence, only departures at times 2, 3, and 4 can forward source symbols to *a* by the deadline. Equivalently, the path has total transit time 3 and bottleneck capacity 1, so it can deliver at most ${\left[5-3+1\right]}_{+}\times 1=3$ symbols by time 5. Transit times determine the usable transmission window, while the path bottleneck determines the delivery rate. In general networks, these effects must be considered jointly across multiple paths and intermediate nodes.

Intermediate nodes may store received symbols and perform causal network coding. Network coding is used only as an upper bound on transport capability. Therefore, infeasibility under coding implies infeasibility under any routing, copying, or replication mechanism satisfying the same arc constraints.

Undirected experimental links are represented by independent antiparallel arcs with equal parameters and therefore model full-duplex communication. At deadline *H*, the observed set identifies nodes known to possess the complete message; no reception status is assigned to the remaining nodes.

**Definition** **1** (Observed Recipients and Candidate-Source Domain)**.** *Let A⊆V denote the set of nodes known to possess the complete K-symbol message by deadline H. No reception status is assigned to nodes in V∖A. Let C⊆V denote the candidate-source domain, namely, the set of nodes considered as possible sources.*

The set *A* records confirmed complete-message reception rather than a complete snapshot of all nodes that possess the message. A node in V∖A may have received the complete message, received only part of it, stored symbols temporarily, or relayed information without being directly observed.

The candidate-source domain C is specified independently of the observed recipient set. When no prior information restricts the source location, all nodes are considered and C=V. When external knowledge guarantees that the source is among the observed nodes, the search may instead be restricted to C=A. More generally, any externally justified subset of *V* may be used as C.

The delivery obligation depends on the candidate being evaluated because a source already possesses the message at time zero.

**Definition** **2** (Required Recipient Set)**.** *For candidate s∈C, the required recipient set is*

(5) $$A_{s}:=A\setminus \left\{s\right\}.$$

*It contains the observed recipients whose complete-message reception must be explained when s is treated as the source.*

The removal of *s* prevents the candidate from being required to transmit the message to itself. If $A_{s}=⌀$, no recipient imposes a delivery requirement. The precise retained and rejected sets, including this boundary case and K=0, are defined after the temporal transport quantity is introduced.

To test candidate *s*, the temporal network is represented by $G_{H}$, the finite-time cut value is evaluated for each $a\in A_{s}$, and *s* is retained only when every required recipient can receive all *K* symbols.

### 3.2. Time-Expanded Network and Exact Retained Set

Following the standard time-expansion construction for flows over time [2,26], we represent the temporal transmission process by a static layered network.

**Definition** **3** (Time-Expanded Network)**.** *For deadline H, define the time-expanded node set*

(6) $$V_{H}:=V\times T_{H}.$$

*Its transmission-arc set is*

(7) $$E_{H}^{\mathrm{tr}}:=\left\{\left(\left(u,t\right),\left(v,t+\tau_{e}\right)\right):e=\left(u,v\right)\in E,\;0\leq t\leq H-\tau_{e}\right\},$$

*and its storage-arc set is*

(8) $$E_{H}^{\mathrm{st}}:=\left\{\left((v,t),(v,t+1)\right):v\in V,\;0\leq t<H\right\}.$$

*The time-expanded network is*

(9) $$G_{H}:=\left(V_{H},E_{H}^{\mathrm{tr}}\cup E_{H}^{\mathrm{st}}\right).$$

*A transmission arc copied from e has capacity $c_{e}$. Every storage arc has capacity*

(10) $$M_{H}:=1+\sum_{e\in E}c_{e}{\left[H-\tau_{e}+1\right]}_{+}.$$

The storage capacity is chosen to be strictly larger than the total capacity of all transmission-arc copies in the expansion. Indeed,

(11) $$\sum_{f\in E_{H}^{\mathrm{tr}}}c_{f}=\sum_{e\in E}c_{e}{\left[H-\tau_{e}+1\right]}_{+}=M_{H}-1.$$

**Figure 2:**
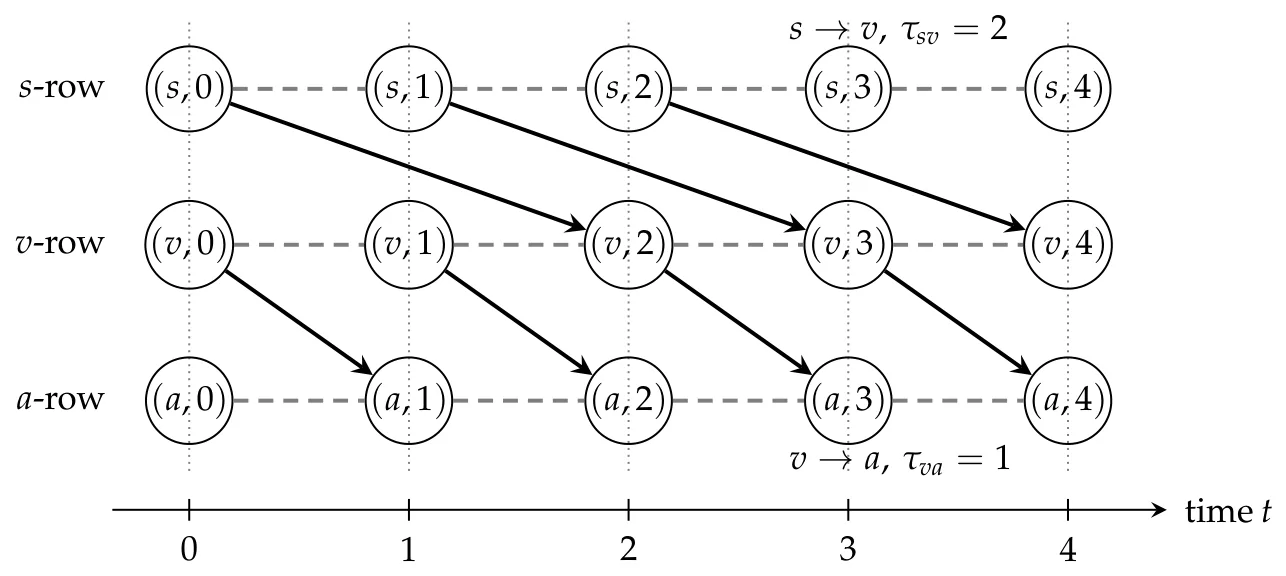
Time-expanded network for the three-node topology with deadline H=4. Each column is a time layer. The dashed same-row segments represent storage from time *t* to time t+1 at the same node. In the formal graph $G_{H}$, these are directed storage arcs, but they are drawn without arrowheads here to distinguish waiting from physical transmission. The diagonal arrows represent transmission arcs. An s→v transmission advances two time layers, whereas a v→a transmission advances one time layer.

Every source–recipient flow is bounded above by this total transmission capacity, so the corresponding minimum-cut value is at most $M_{H}-1$. Any cut that severs even one storage arc has a capacity of at least $M_{H}$ and therefore cannot be a minimum. Storage arcs consequently preserve symbols across time layers without becoming bottlenecks; their finite value is only a convenient replacement for infinite capacity. Because every arc advances time strictly, $G_{H}$ is acyclic. Figure 2 illustrates the construction using the same three-node topology as Figure 1. An earlier deadline H=4 is used only to keep the layered representation compact.

A path in the time-expanded network records both when information is transmitted and when it is stored. For example, the path

(s,0)→(v,2)→(a,3)

represents information that is transmitted from *s* at time 0, reaches *v* at time 2, and is immediately forwarded to *a*, where it arrives at time 3. If forwarding is delayed, the path instead uses one or more storage arcs such as

(v,2)→(v,3).

Thus, reaching (a,H) means that the information has arrived at node *a* no later than the deadline, with any earlier arrival carried forward through storage arcs to the final time layer.

**Definition** **4** (Pairwise Temporal Min-Cut)**.** *For distinct s,a∈V, let $\delta_{H}^{+}\left(X\right)$ denote the arcs of $G_{H}$ leaving a set $X\subseteq V_{H}$, and let $c_{H}\left(F\right)$ be the total capacity of an arc set F. Define*

(12) $$\begin{array}{cc} \lambda_{H}\left(s,a\right)\; & :={\mathrm{mincut}}_{G_{H}}\left((s,0),(a,H)\right)\\ & :=\min_{\begin{array}{c} X\subseteq V_{H}:\\ (s,0)\in X,\;(a,H)\notin X \end{array}}c_{H}\left(\delta_{H}^{+}\left(X\right)\right). \end{array}$$

Thus, $\lambda_{H}\left(s,a\right)$ is the smallest total capacity that separates the candidate at time zero from recipient *a* at the deadline. The storage arcs ensure that reaching (a,H) is equivalent to reaching *a* at any time no later than *H*.

**Definition** **5** (Candidate Delivery Value and Exact Retained Set)**.** *For candidate s∈C, define*

(13) $$\Phi_{H}\left(s;A\right):=\left\{\begin{array}{cc} \min_{a\in A_{s}}\lambda_{H}\left(s,a\right), & A_{s}\neq ⌀,\\ +\infty , & A_{s}=⌀. \end{array}\right.$$

*The exact retained candidate set is*

(14) $$S_{H}^{\mathrm{exact}}\left(K,A,C;c,\tau \right):=\left\{s\in C:\Phi_{H}\left(s;A\right)\geq K\right\}.$$

*When c and τ are fixed, they are suppressed from the notation. A candidate in* $S_{H}^{\mathrm{exact}}$ *is called* retained, *and every other candidate is called* rejected.

The candidate value $\Phi_{H}\left(s;A\right)$ is governed by the required recipient with the smallest pairwise temporal min-cut. Retention is therefore a feasibility statement under the coded transport model, not a likelihood or ranking statement.

**Theorem** **1** (Exact finite-time candidate characterization)**.** *Over a sufficiently large finite field, candidate s can deliver the same K-symbol message to every node in $A_{s}$ by deadline H using causal network coding if and only if*

(15) $$\Phi_{H}\left(s;A\right)\geq K.$$

*Equivalently, candidate s is retained according to Definition 5 if and only if this coded-delivery condition holds.*

**Proof.**  See Appendix A. □

**Theorem** **2** (Conservative Safety of Rejection)**.** Suppose all K message symbols are initially available at a true source $s^{*}\in C$, and a causal protocol respecting the modeled capacities and transit times delivers the complete message to every node in $A_{s^{*}}$ by deadline H. Then

(16) $$s^{*}\in S_{H}^{\mathrm{exact}}\left(K,A,C\right).$$

*The protocol need not use network coding.*

**Proof.**  See Appendix B. □

Theorem 2 explains the one-sided interpretation. If $s\notin S_{H}^{\mathrm{exact}}$, then even ideal network coding fails, so routing or replication also fails. If $s\in S_{H}^{\mathrm{exact}}$, the candidate is coded as feasible under the stated model but may still fail under a more restrictive prescribed protocol.

### 3.3. Monotonicity and Interval Uncertainty

The retained set is monotone under larger capacities, shorter transit times, a later deadline, a smaller payload, or fewer required recipients.

**Theorem** **3** (Monotonicity of the Exact Retained Set)**.** *Consider two instances on the same directed graph and candidate domain. Suppose $c_{e}^{\prime }\geq c_{e}$ and $\tau_{e}^{\prime }\leq \tau_{e}$ for every e∈E, and suppose $H^{\prime }\geq H$, $K^{\prime }\leq K$, and $A^{\prime }\subseteq A$. Then,*

(17) $$S_{H}^{\mathrm{exact}}\left(K,A,C;c,\tau \right)\subseteq S_{H^{\prime }}^{\mathrm{exact}}\left(K^{\prime },A^{\prime },C;c^{\prime },\tau^{\prime }\right).$$

**Proof.**  See Appendix C. □

Suppose now that the payload, deadline, capacities, and transit times are known only through integer intervals

(18) $$K\in \left[K_{-},K_{+}\right],\;H\in \left[H_{-},H_{+}\right],\;c_{e}\in \left[c_{e}^{-},c_{e}^{+}\right],\;\tau_{e}\in \left[\tau_{e}^{-},\tau_{e}^{+}\right].$$

All interval endpoints are integers with $c_{e}^{-}\geq 1$ and $\tau_{e}^{-}\geq 1$, so every admissible realization satisfies the standing model assumptions. The two endpoint instances induce the nested sets

(19) $$\begin{array}{cc} S^{\mathrm{fav}} & :=S_{H_{+}}^{\mathrm{exact}}\left(K_{-},A,C;c^{+},\tau^{-}\right), \end{array}$$

(20) $$\begin{array}{cc} S^{\mathrm{unfav}} & :=S_{H_{-}}^{\mathrm{exact}}\left(K_{+},A,C;c^{-},\tau^{+}\right), \end{array}$$

where Theorem 3 gives

(21) $$S^{\mathrm{unfav}}\subseteq S^{\mathrm{fav}}.$$

**Definition** **6** (Candidate Status under Interval Uncertainty)**.** *The candidate domain is partitioned into*

(22) $$\begin{array}{cccc} R^{\mathrm{def}} & :=C\setminus S^{\mathrm{fav}},\; & & \mathrm{definitely}\;\mathrm{rejected}\;\mathrm{candidates}, \end{array}$$

(23) $$\begin{array}{ccc} F^{\mathrm{all}}\; & :=S^{\mathrm{unfav}},\; & \mathrm{candidates}\;\mathrm{coded}-\mathrm{feasible}\;\mathrm{throughout}\;\mathrm{the}\;\mathrm{range}, \end{array}$$

(24) $$\begin{array}{ccc} I\; & :=S^{\mathrm{fav}}\setminus S^{\mathrm{unfav}},\; & \mathrm{indeterminate}\;\mathrm{candidates}. \end{array}$$

**Corollary** **1** (Endpoint Certificates under Interval Uncertainty)**.** *Every candidate in $R^{\mathrm{def}}$ is rejected for every admissible parameter realization, and every candidate in $F^{\mathrm{all}}$ satisfies the coded-delivery condition for every admissible realization. The endpoint tests make no uniform conclusion for candidates in I.*

**Proof.**  See Appendix D. □

## 4. Exact Computation and Staged Candidate Elimination

### 4.1. Minimum-Cost Circulation Computation

The time-expanded network defines $\lambda_{H}\left(s,a\right)$, but its size grows with *H*. For distinct s,a∈V, assign each original arc *e* capacity $c_{e}$ and cost $\tau_{e}$, and add a return arc a→s with cost −(H+1) and capacity

(25) $$C_{s}^{\mathrm{out}}:=\sum_{e\in \delta^{+}\left(s\right)}c_{e}.$$

Let $C_{s,a,H}^{*}$ be the minimum circulation cost in this augmented network.

**Proposition** **1** (Exact Computation by Minimum-Cost Circulation)**.** *For distinct s,a∈V,*

(26) $$\lambda_{H}\left(s,a\right)=-C_{s,a,H}^{*}.$$

This is the Ford–Fulkerson maximum-flow-over-time reduction. The equivalent static-flow optimization and the proof are given in Appendix E.

The exact test is now fully specified, but it costs one circulation per candidate–recipient pair. The next subsection develops cheaper certificates that reject many candidates before any exact computation is needed.

### 4.2. Safe Preliminary Screens

When K=0, every candidate is retained, and no screening is needed. Assume throughout this subsection that K>0. Exact evaluation may require one pairwise computation for every (s,a) with s∈C and $a\in A_{s}$. The following upper bounds reject some candidates before Proposition 1 is invoked.

Define

(27) $$D_{\tau }\left(s;A\right):=\left\{\begin{array}{cc} \min_{a\in A_{s}}d_{\tau }\left(s,a\right), & A_{s}\neq ⌀,\\ 0, & A_{s}=⌀, \end{array}\right.$$

and the shortest-transit retained set

(28) $$S_{H}^{\mathrm{delay}}\left(A,C\right):=\left\{s\in C:D_{\tau }\left(s;A\right)\leq H\right\}.$$

The static source-candidate aggregation follows the earlier rate-feasibility formulation [1], but it is used here only as an upper-bound screen.

**Definition** **7** (Static-Capacity Screen)**.** *For distinct s,a∈V, define the ordinary static minimum cut*

(29) $$\lambda \left(s,a\right):=\min_{\begin{array}{c} B\subseteq V:\\ s\in B,\;a\notin B \end{array}}\sum_{e\in \delta^{+}\left(B\right)}c_{e}.$$

*For candidate s, define*

(30) $$\rho \left(s;A\right):=\left\{\begin{array}{cc} \min_{a\in A_{s}}\lambda \left(s,a\right), & A_{s}\neq ⌀,\\ +\infty , & A_{s}=⌀, \end{array}\right.$$

*and*

(31) $$S_{H}^{\mathrm{static}}\left(K,A,C\right):=\left\{s\in C:H\rho \left(s;A\right)\geq K\right\}.$$

**Proposition** **2** (Preliminary Screening Inclusions)**.** *For every distinct s,a∈V,*

(32) $$d_{\tau }\left(s,a\right)>H\;⟹\;\lambda_{H}\left(s,a\right)=0,\;\lambda_{H}\left(s,a\right)\leq H\lambda \left(s,a\right).$$

*Consequently,*

(33) $$S_{H}^{\mathrm{exact}}\left(K,A,C\right)\subseteq S_{H}^{\mathrm{delay}}\left(A,C\right)\cap S_{H}^{\mathrm{static}}\left(K,A,C\right).$$

**Proof.**  See Appendix F. □

The factor *H* in (32) depends on the standing assumption $\tau_{e}\geq 1$. If zero transit times were allowed, the correct factor would be H+1. The two screens capture different obstructions. The shortest-transit set tests whether every required recipient can be reached at all by the deadline, whereas the static-capacity set bounds the total amount that can cross a static bottleneck during at most *H* usable slots.

Appendix G defines a deadline-adjusted static cut and proves the intermediate hierarchy

(34) $$S_{H}^{\mathrm{exact}}\subseteq S_{H}^{\mathrm{adjusted}}\subseteq S_{H}^{\mathrm{static}}.$$

This bound is included in the numerical comparison, although it produced negligible additional rejection.

**Figure 3:**
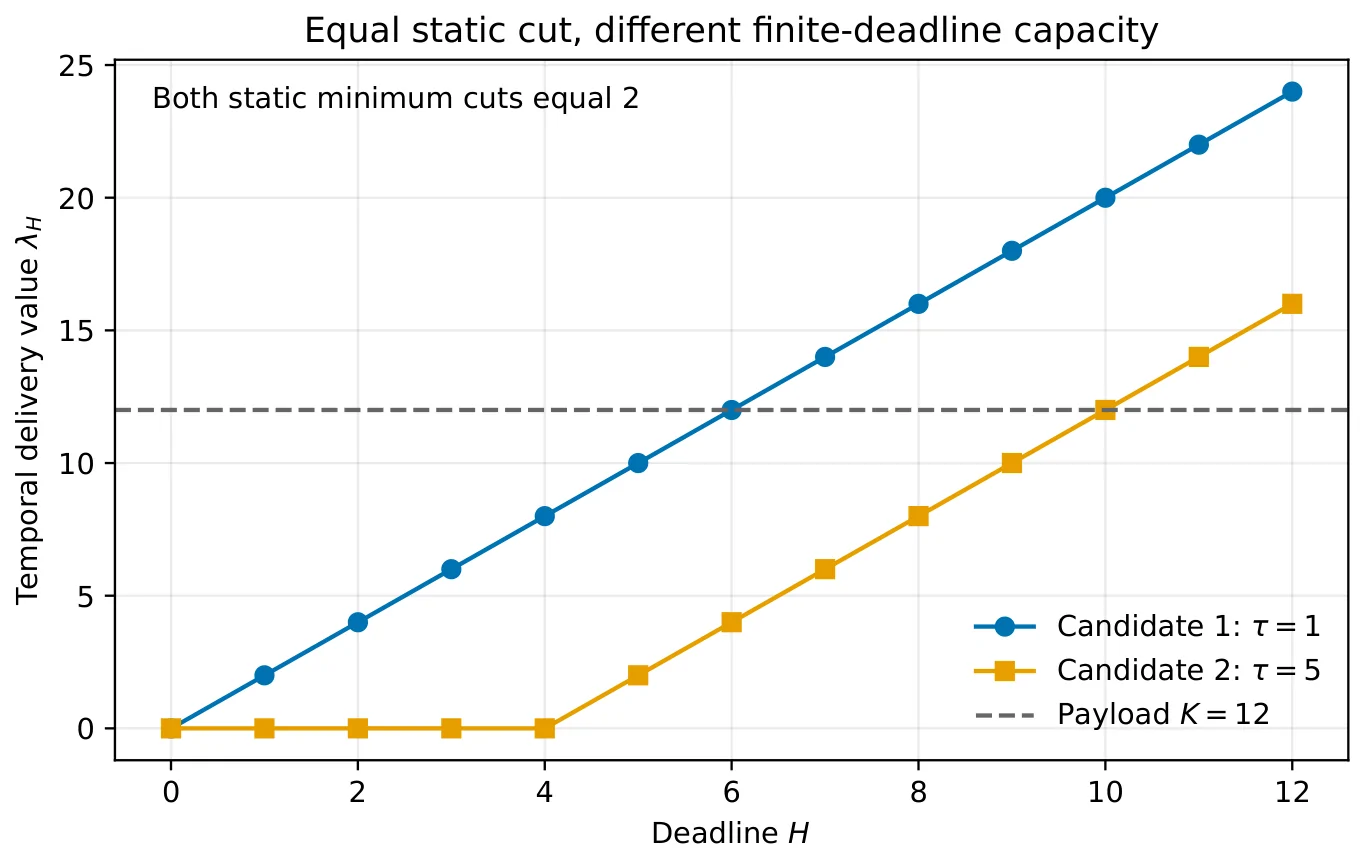
Equal static minimum cuts do not imply equal finite-time delivery values. Each single-path candidate has a capacity of two, while its transit time changes the number of usable departure slots.

Figure 3 gives the smallest illustrative separation between static and finite-time feasibility. Both candidates have a static minimum cut of two to the recipient, but their transit times are one and five. With payload K=12, the first candidate reaches the threshold at H=6, whereas the second requires H=10. Static capacity alone cannot distinguish them.

### 4.3. Staged Candidate Evaluation

Exact evaluation may stop as soon as one required recipient satisfies $\lambda_{H}\left(s,a\right)<K$. Passing only a subset of recipients does not certify the candidate. Let TemporalCut(s,a,H) denote the value obtained from the minimum-cost circulation in Proposition 1. Algorithm 1 applies the safe screens first and evaluates exact pairwise values only for candidates that survive them. The optional deadline-adjusted stage may be omitted without changing the final retained set. The recipient order $\pi_{s}$ affects only early stopping.


**Algorithm 1:** Staged exact elimination of source candidates.

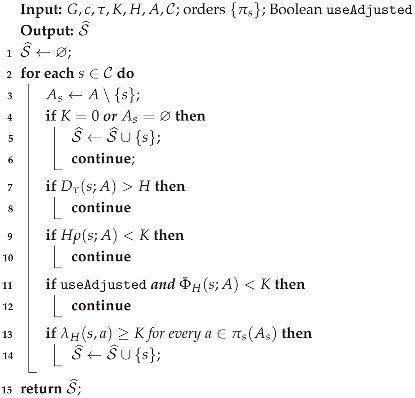




Every preliminary rejection in Algorithm 1 follows from Proposition 2 or the deadline-adjusted hierarchy, and the final stage applies the exact pairwise condition. An implementation may additionally record an attaining recipient and bound value as a rejection certificate. Exact size formulas for the time-expanded network are recorded in Appendix H. The screens apply to arbitrary directed networks. On trees, the exact value itself has a closed form, which the next section turns into all-candidate algorithms.

### 4.4. Complexity and Recipient Ordering

Let *n*, *m*, *q*, and *r* denote the numbers of nodes, arcs, candidate sources, and the maximum number of active observed recipients. Let $T_{\mathrm{sp}}\left(n,m\right)$, $T_{\mathrm{mf}}\left(n,m\right)$, and $T_{\mathrm{mcc}}\left(n,m\right)$ denote the costs of one nonnegative shortest-path computation, one maximum-flow computation, and one minimum-cost circulation computation. With all screening stages enabled and recipients sorted for early stopping, the worst-case running time of Algorithm 1 is

(35) $$O\;\left(q\left[T_{\mathrm{sp}}\left(n,m\right)+r\log r+r\left(2T_{\mathrm{mf}}\left(n,m\right)+T_{\mathrm{mcc}}\left(n,m+1\right)\right)\right]\right).$$

The two maximum-flow terms correspond to the static and optional deadline-adjusted cuts. Omitting the adjusted stage removes one term. The exact minimum-cost circulation oracle uses the *n* original nodes and m+1 augmented arcs before residual reverse-arc bookkeeping. Sequential candidate and recipient processing requires $O(n+m+r+S_{\mathrm{oracle}})$ live working space, where $S_{\mathrm{oracle}}$ is the largest active shortest-path or flow-solver workspace. Explicit time expansion instead uses n(H+1) nodes and $nH+\sum_{e}{\left[H-\tau_{e}+1\right]}_{+}$ arcs before solver-specific overhead. Preliminary rejection and early stopping reduce observed work but do not improve the worst-case bound.

Recipient ordering affects only the time at which an already-infeasible candidate is discovered. It cannot change the retained set because every active recipient must pass. A practical order places recipients in descending shortest-transit time and uses values already computed by the preliminary screen. The comparative ordering experiment and its operation-count results appear in Section 7.2 and Supplementary Section S2.

## 5. Tree Characterizations and All-Candidate Algorithms

On trees, the temporal value admits a closed-form expression. Assume that the underlying undirected topology is a tree and that each physical link is represented by two opposite arcs with the same positive capacity and transit time.

**Definition** **8** (Tree Path Parameters)**.** *For distinct nodes s and a, let $P_{sa}$ be their unique undirected path, oriented from s to a. Its bottleneck capacity is*

(36) $$b_{sa}:=\min_{e\in P_{sa}}c_{e}.$$

*Because the tree is bidirected and $P_{sa}$ is the unique path, (2) reduces to*

(37) $$d_{\tau }\left(s,a\right)=\sum_{e\in P_{sa}}\tau_{e}.$$

### 5.1. Pairwise Capacity and Candidate Completion Time

**Theorem** **4** (Finite-Time Capacity on a Tree Path)**.** *For distinct nodes s and a,*

(38) $$\lambda_{H}\left(s,a\right)=b_{sa}{\left[H-d_{\tau }\left(s,a\right)+1\right]}_{+}.$$

**Proof.**  See Appendix I. □

**Corollary** **2** (Earliest Candidate Completion Time on a Tree)**.** *For K=0 or $A_{s}=⌀$, define Θ(s;A,K):=0. Otherwise,*

(39) $$\Theta \left(s;A,K\right):=\min_{a\in A_{s}}\left\{d_{\tau }\left(s,a\right)-1+\left\lceil \frac{K}{b_{sa}}\right\rceil \right\}.$$

*Then Θ(s;A,K) is the smallest deadline for which candidate s is retained, and*

(40) $$s\in S_{H}^{\mathrm{exact}}\left(K,A,C\right)\;⟺\;\Theta \left(s;A,K\right)\leq H.$$

**Proof.**  The proof is included with Theorem 4 in Appendix I. □

**Corollary** **3** (Static Degeneracy on Unit-Capacity Trees)**.** *Suppose every link has unit capacity and unit transit time, and $A_{s}\neq ⌀$. Then ρ(s;A)=1 for every candidate s in the connected tree, while*

(41) $$\Theta \left(s;A,K\right)=K-1+\min_{a\in A_{s}}d_{\mathrm{hop}}\left(s,a\right),$$

*where $d_{\mathrm{hop}}$ is the ordinary hop distance. Thus the static screen makes the same decision for all candidates, whereas the exact finite-time test remains source-dependent.*

**Proof.**  See Appendix J. □

### 5.2. Uniform Capacities and Linear-Time Evaluation

Suppose every link has a common capacity c>0 but may have a different transit time. The shortest-transit distance $d_{\tau }$ is then a weighted tree metric. Define

(42) $$r_{H}:=H+1-\left\lceil \frac{K}{c}\right\rceil .$$

For a node *a* and radius *r*, write

(43) $$B_{\tau }\left(a,r\right):=\left\{v\in V:d_{\tau }\left(v,a\right)\leq r\right\}.$$

**Theorem** **5** (Retained-set geometry on a uniform-capacity tree)**.** *Let |A|≥2 and K>0. Then*

(44) $$S_{H}^{\mathrm{exact}}\left(K,A,C\right)=C\cap \underset{a\in A}{⋂}B_{\tau }\left(a,r_{H}\right).$$

*In particular, the retained set is a singleton exactly when the right-hand side contains one candidate.*

**Proof.**  See Appendix K. □

When the retained set is a singleton and the observation was in fact produced by a model-consistent transmission from a source in C, Theorem 2 implies that the singleton candidate is the true source.

**Figure 4:**
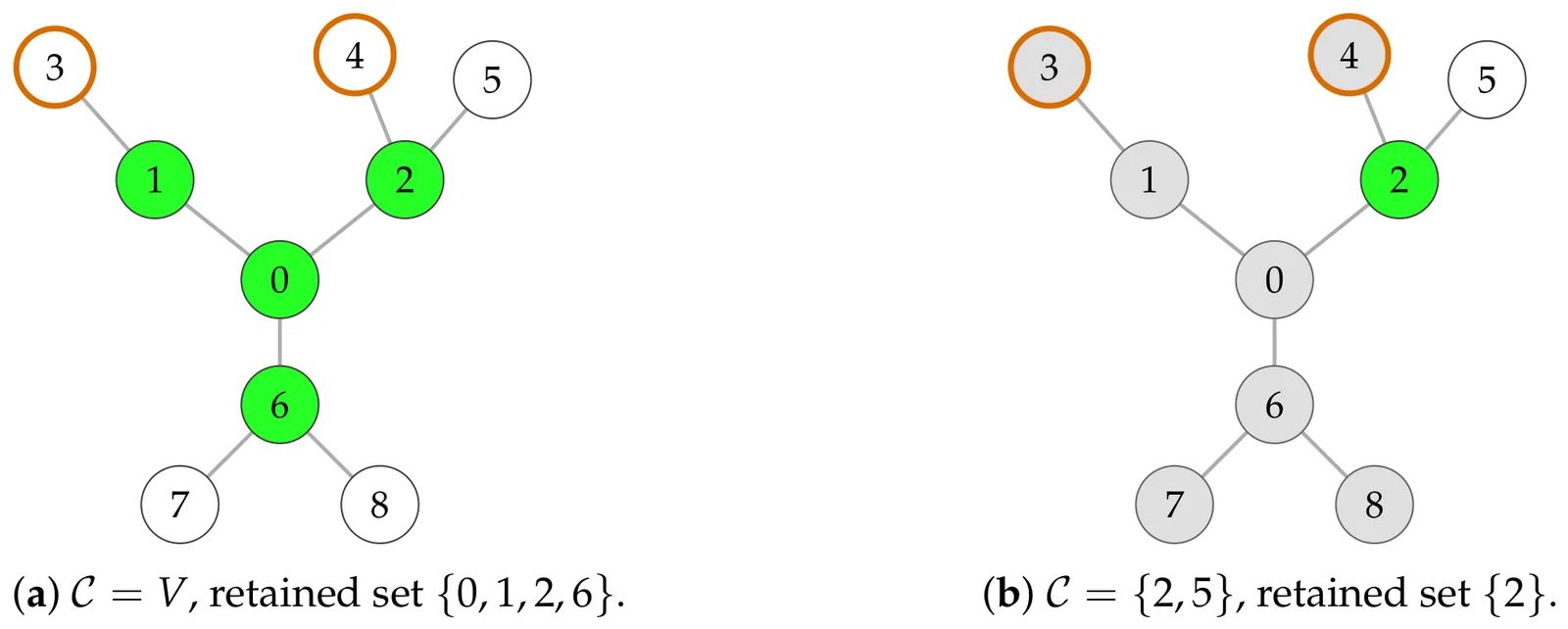
Retained-set geometry on a uniform-capacity tree with unit transit times, observed set A={3,4}, and radius $r_{H}=3$. The two recipient-centered balls intersect in {0,1,2,6}. In panel (**a**), C=V, so all four nodes in the intersection are retained. In panel (**b**), C={2,5}, yielding the singleton retained set {2}. Green nodes are retained, white nodes belong to the candidate domain but are rejected, gray nodes lie outside the candidate domain, and orange borders identify observed recipients.

Figure 4 illustrates Theorem 5 on a tree with unit transit times. Let A={3,4} and $r_{H}=3$. The recipient-centered balls satisfy

$$B_{\tau }\left(3,3\right)=\left\{0,1,2,3,6\right\},\;B_{\tau }\left(4,3\right)=\left\{0,1,2,4,5,6\right\},$$

so their intersection is {0,1,2,6}. When C=V, all four nodes are retained. Under the external restriction C={2,5}, only node 2 remains in the exact retained set; the transport-feasible ball intersection itself is unchanged.

Let p,q∈A be endpoints of the diameter of the observed set under $d_{\tau }$. The terminal-diameter identity proven in Appendix L gives

(45) $$\Theta \left(v;A,K\right)=\left\lceil \frac{K}{c}\right\rceil -1+\min\left\{d_{\tau }\left(v,p\right),d_{\tau }\left(v,q\right)\right\}.$$

Thus, two weighted-tree traversals after finding *p* and *q* determine all candidate completion times in O(|V|) time and O(|V|) space. When |A|=1, the observed node has a completion time of zero when evaluated as the candidate, and every other candidate uses its distance to that recipient.

### 5.3. Heterogeneous Capacities and All-Candidate Evaluation

With heterogeneous capacities, the term $\left\lceil K/b_{va}\right\rceil$ depends jointly on the endpoints, so the diameter reduction no longer applies. A centroid decomposition recursively separates the tree into components of at most half the current size [32]. Its formal definition and the branch-excluding query construction are given in Appendix M.

**Theorem** **6** (All-candidate completion on a heterogeneous tree)**.** *For any nonempty observed set A and integer payload K≥0, all values Θ(v;A,K) in (39) can be computed in*

(46) $$O\left(\left|V\right|\log^{2}\left|V\right|\right)$$

*time and O(|V|) working space.*

**Proof.**  See Appendix M. □

For a centroid *z* of the current component *U*, let $\ell_{z}\left(u\right)=d_{\tau }\left(z,u\right)$, let $\beta_{z}\left(u\right)$ be the minimum capacity on $P_{zu}$, and let $\gamma_{z}\left(u\right)$ be the branch of U−z containing *u*. We set $\ell_{z}\left(z\right)=0$, $\beta_{z}\left(z\right)=+\infty$, use the convention ⌈K/(+∞)⌉=0, and assign *z* its own branch label. Only recipients in A∩U enter the query at this component. For branch-labeled values, TopTwo retains the two largest values contributed by distinct branches, and ${\mathrm{best}}_{\neq g}$ returns the largest stored value whose branch differs from *g*, or −∞ if none exists. Algorithm 2 states the all-candidate procedure.

At each centroid, the prefix class contains recipients with $\beta_{z}\left(a\right)<\beta_{z}\left(v\right)$ and therefore stores $\ell_{z}\left(a\right)+\left\lceil K/\beta_{z}\left(a\right)\right\rceil$; the suffix class contains recipients with $\beta_{z}\left(a\right)\geq \beta_{z}\left(v\right)$ and therefore stores only $\ell_{z}\left(a\right)$ before adding the candidate-dependent term $\left\lceil K/\beta_{z}\left(v\right)\right\rceil$. A lower-bound search places ties $\beta_{z}\left(a\right)=\beta_{z}\left(v\right)$ in the suffix. Branch-distinct maxima exclude recipients whose path to the candidate does not pass through the current centroid, while the recursion processes same-branch pairs in the corresponding child component. Consequently, $\hat{\Theta }\left(v\right)$ can remain −∞ only when A∖{v}=⌀.
**Algorithm 2:** All-candidate completion times on a heterogeneous tree.
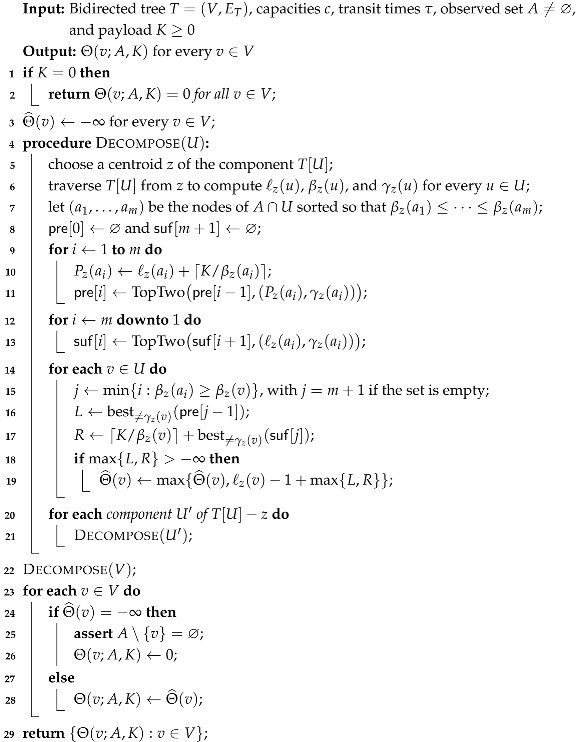


For a direct comparison, let n=|V| and r=|A|. A bidirected tree contains 2(n−1) directed transmission arcs. By Appendix H, its generic time-expanded representation has

(47) $$|V_{H}\left|=n\left(H+1\right),\;\right|E_{H}|=nH+2\sum_{\left\{u,v\right\}\in E_{T}}{\left[H-\tau_{uv}+1\right]}_{+}=O\left(nH\right).$$

Using the maximum-flow cost $T_{\mathrm{mf}}$ defined in Section 4.4, evaluating every candidate against every required recipient by the generic time-expanded method therefore takes

(48) $$O\;\left(nH+nr\;T_{\mathrm{mf}}\;\left(n\left(H+1\right),nH+2\sum_{\left\{u,v\right\}\in E_{T}}{\left[H-\tau_{uv}+1\right]}_{+}\right)\right)$$

time and requires O(nH) expanded-graph storage before solver-specific workspace. When r=Θ(n), the method performs $\Theta (n^{2})$ pairwise flow computations on a graph whose size grows linearly with *H*. Independent evaluation of the closed-form tree expression avoids time expansion but still requires O(nr) pair evaluations and becomes $O(n^{2})$ when r=Θ(n). This is the independent-pairs baseline evaluated in Section 7.2. Centroid decomposition instead exploits unique tree paths and computes all candidate completion times in $O(n\log^{2}n)$ time with O(n) working space. Its asymptotic operation count has no multiplicative dependence on *H*.

## 6. Experimental Program

The experiments progressively characterize the certified finite-time retained set. The sequence begins with controlled validation of safety and selectivity. It then evaluates computational efficiency, transfer beyond calibrated instances, behavior on real network topologies, fidelity of the real-network representation, and the dependence of selectivity on the permitted transmission protocol. Each later study therefore tests a stronger form of validity than the study before it.

### 6.1. Experimental Questions and Common Measures

The empirical program addresses six questions. First, does exact finite-time feasibility retain a model-consistent true source while removing candidates that static feasibility cannot reject? Second, can safe screens, recipient ordering, and tree structure reduce the cost of exact certification? Third, does refinement transfer to networks that were not used to choose an operating configuration? Fourth, does the same behavior persist on real backbone topologies? Fifth, does the normalization used for those topologies preserve the shortest-path geometry that drives temporal screening? Sixth, when is the coded retained set narrow, and when is it naturally permissive under an actual protocol?

For candidate *s*, let $A_{s}=A\setminus \left\{s\right\}$ denote the active recipient set. The static and exact retention fractions are

(49) $$r_{\mathrm{static}}=\frac{|S_{H}^{\mathrm{static}}|}{\left|C\right|},\;r_{\mathrm{exact}}=\frac{|S_{H}^{\mathrm{exact}}|}{\left|C\right|}.$$

When the static set is nonempty, its relative refinement is

(50) $$R_{\mathrm{set}}=\frac{|S_{H}^{\mathrm{static}}\left|-\right|S_{H}^{\mathrm{exact}}|}{|S_{H}^{\mathrm{static}}|}.$$

When a true source is known, two additional measures are used. The false-elimination rate records whether a model-consistent source is rejected, while false-candidate refinement removes the true source from both retained sets before measuring the reduction.

(51) $$\mathrm{FER}=\frac{1}{N}\sum_{i=1}^{N}1\;\left\{s_{i}^{*}\notin S_{H,i}^{\mathrm{exact}}\right\}.$$

(52) $$R_{\mathrm{false}}=\frac{|S_{H}^{\mathrm{static}}\setminus \left\{s^{*}\right\}\left|-\right|S_{H}^{\mathrm{exact}}\setminus \left\{s^{*}\right\}|}{|S_{H}^{\mathrm{static}}\setminus \left\{s^{*}\right\}|}.$$

A zero denominator makes the corresponding refinement measure inapplicable. Controlled observations without a generated source are used only to study retained-set selectivity. Protocol-generated observations are used to evaluate safety.

The independent unit follows the level at which new networks or transmission realizations are generated. Protocol studies use the base network. Held-out calibration uses the unseen test tree. GEANT2009 uses the scenario. The broad real-network study first averages five scenarios within each family and then treats the 40 network families as the primary units. The RLNC diagnostic treats the 100 frozen base graphs as the primary units rather than the 1200 candidates or 153,600 coding trials. Bootstrap intervals resample these primary units [33]. The held-out and recipient-ordering studies use 4000 deterministic repetitions. The matched protocol, GEANT2009, 40-family, and RLNC summaries use 10,000 deterministic repetitions. Table 1 summarizes the experimental sequence, populations, and primary units of analysis.

### 6.2. Experimental Sequence and Frozen Designs

The controlled protocol study contains three regimes. P1 uses 18-node bidirected heterogeneous trees with capacities and transit times in [1,5], payload K=5, and decode-and-replicate transmission. P2 uses 14-node directed graphs formed from a directed cycle and additional arcs sampled with probability 0.24. Its capacities lie in [1,5], transit times lie in [1,4], and K=5. For P1 and P2, the deadline is the 0.45 quantile of non-source completion times and the observed set contains the non-source nodes completed by that time. P3 uses 12-node directed graphs with additional-arc probability 0.28, capacities in [1,4], transit times in [1,3], payload K=4, and deadline H=8. Each arc sends causal random linear combinations over $F_{257}$ in every slot. Nodes with rank four at *H* form the observed set. All regimes require at least four observed recipients.

Each realization is analyzed under the full domain (A,C=V) and the source observed domain

$$A^{+}=A\cup \left\{s^{*}\right\},\;C=A^{+}.$$

The two analyses remain paired because they share the same generated graph and transmission. The source-observed analysis changes both the observed set and the candidate domain.

The controlled tree study uses 100 paired 64-node trees. Each tree contains a 16-node core and eight peripheral branches. Four parameter conditions compare uniform and heterogeneous capacities with uniform and heterogeneous transit times on the same topology. Recipient placement, observed-set size, and bounded joint uncertainty are varied through paired designs. These observations have no generated source and therefore measure selectivity rather than safety.

The original calibration study chooses a configuration separately on each of 100 trees for four target static-retention levels. The held-out study separates selection from evaluation. It selects one global configuration per target by using only static-screen results on 100 calibration trees. It then freezes the location, payload, and deadline slack before evaluation on 200 newly generated trees with disjoint realized topologies. No exact retained set or temporal oracle is consulted during selection.

The computational studies compare the minimum-cost circulation formulation with explicit time expansion, profile the safe screening stages, compare centroid decomposition with independent tree-path evaluation, and test five recipient orders. The descending shortest-transit order is selected in this isolated ordering study and is frozen before the real-network evaluations.

The detailed real-network study uses GEANT2009 with 34 nodes and 52 physical links represented by 104 directed arcs. Nominal link rates come from the Internet Topology Zoo and benchmark delays come from REPETITA. Capacity and delay discretizations are selected from structural analyses before source evaluation. Two hundred decode-and-replicate scenarios with only positive recipient observations use K=32, a deadline set by the tenth non-source completion, and eight sampled eligible recipients. Scenario 0 is fixed in advance as the illustrative case.

The broad transfer study uses the most recent eligible REPETITA snapshot from each of 40 Internet Topology Zoo families. The networks contain 50 to 197 nodes. Self-loops are removed, parallel arcs are preserved, and each network maximum is mapped to 32 capacity units and 64 transit-time units. Five scenarios with only positive recipient observations are fixed per network with K=256. This produces 200 scenarios and 16,960 candidate evaluations. A separate exact-arithmetic audit evaluates the shortest-distance and shortest-path effects of the delay normalization independently of source-identification outcomes.

The RLNC mechanism diagnostic reuses the 100 frozen P3 graphs with the same *K*, *H*, field, and graph population. It runs 128 deterministic coding trials for every graph and candidate pair. The recorded rank and innovation dynamics are compared with exact coded feasibility and with a decode-before-forward counterfactual.

### 6.3. Implementation and Independent Verification

The reference implementation contains directed maximum flow, static and deadline-adjusted cuts, exact original-network minimum-cost computation, an independent time-expanded computation, selected-recipient early stopping, staged candidate evaluation, tree formulas, and two all-candidate tree algorithms. A retained candidate means only that no rejection certificate fired. The implementation does not interpret retention as protocol feasibility.

Independent numerical checks compare the original-network and time-expanded exact methods on 1200 random directed instances. They check the tree formula on 1600 path and deadline cases and compare centroid decomposition with direct candidate–recipient traversal on 2000 heterogeneous trees. Further tests cover exhaustive cuts, residual rerouting, candidate self-exclusion, zero payload, empty active recipient sets, parallel arcs, self-loops, and isolated-stage profiling.

The observation generators are independent of the screening computation. Decode-and-replicate generators forward only after complete decoding. The coded generator processes arrivals before transmissions in each slot and transmits causal random linear combinations subject to every arc capacity and transit time. Frozen policies, deterministic seeds, artifact hashes, and independent replay audits protect the later studies from outcome-based replacement or retuning. The real-network audits reconstruct preprocessing, scenarios, candidate decisions, aggregation, and bootstrap intervals. The RLNC audit independently replays every prescribed coding trial. Supplementary definitions and complete secondary results are provided in Supplementary Sections S1–S6.

## 7. Experimental Results

### 7.1. Controlled Validation of Safety and Selectivity

The first question is whether the exact retained set is safe and whether it is tighter than the static set. Table 2 reports the matched protocol results.

None of the 300 independent base-graph trials produced a false elimination. The pooled zero-event count gives a one-sided 95% upper bound of 0.994% for the equal-weight mixture of the three protocol designs. Both decode-and-replicate regimes show substantial refinement. The RLNC regime shows weak refinement even though the safety result remains intact. This contrast establishes that safety and selectivity are different properties. A safe certificate may remain broad when the underlying coded transport model makes many candidates feasible.

The tree formula and original-network exact computation return identical candidate sets throughout P1. The deadline-adjusted cut removes little beyond the static set in these synthetic regimes. Shortest transit time and the static cut provide the main inexpensive rejections, while the exact oracle supplies the remaining finite-time discrimination.

The controlled tree study identifies which transport features shape the retained set. Table 3 compares approximately mean-matched capacity and transit-time conditions for core recipients.

Capacity heterogeneity reduces the mean static count from 64 to 46 and the mean exact count from 16.55 to 8.50. Transit-time heterogeneity reduces the mean shortest-transit count from 62.24 to 58.51 and changes the mean exact count from 16.55 to 16.29. Under joint heterogeneity, recipient placement has an even larger effect. Mean exact counts for core, mixed, and peripheral recipients are 8.93, 0.19, and 0. The result reflects the transport geometry of the tested generator and is not interpreted as a population-wide causal effect.

**Figure 5:**
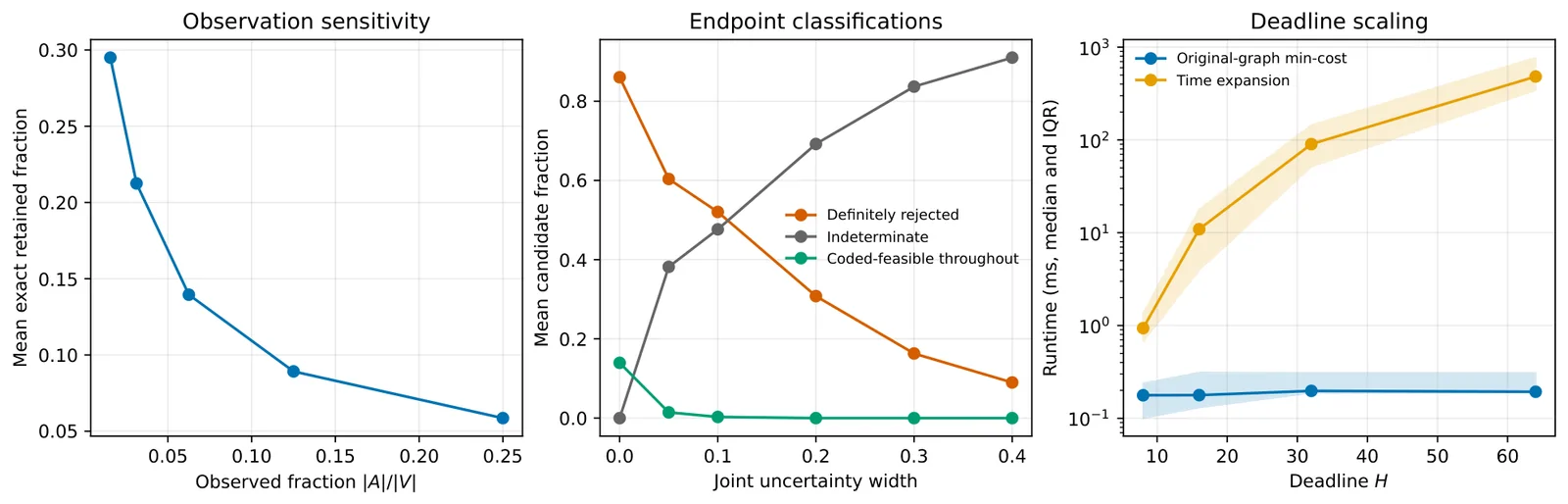
Sensitivity to observed-recipient fraction and joint parameter uncertainty, together with deadline scaling of the exact runtime benchmark. Runtime curves show the pooled median and interquartile range over stored repetitions.

More observed recipients lead to smaller retained sets. For |A|=1,2,4,8,16, the paired mean exact counts are 18.88, 13.60, 8.93, 5.71, and 3.75. This empirical monotonicity agrees with Theorem 3. Wider parameter intervals reduce decisiveness. At zero uncertainty width, the mean counts of definitely rejected, indeterminate, and candidates feasible throughout the uncertainty interval are 55.07, 0, and 8.93. At 40% width, the corresponding counts are 5.76, 58.24, and 0. Figure 5 summarizes the recipient, uncertainty, and deadline sensitivity results.

### 7.2. Computational Efficiency of Certification

The next question is whether exact certification can be made practical. Table 4 isolates the screening stages. Table 5 and Table 6 compare exact formulations and all-candidate tree methods.

All 33 paired directed evaluations return identical exact values. The minimum-cost circulation median remains near 0.2 ms in the fixed-graph horizon sweep, while the time-expanded network runtime grows from 0.937 ms at H=8 to 482.592 ms at H=64. The result agrees with the theoretical difference between an oracle on the original graph and a graph whose size grows with the horizon.

All 31 tree rows return identical completion times. The measured speedup rises from 1.9 at 64 nodes to 45.0 at 2048 nodes. This pattern is consistent with the comparison between independent O(nr) path evaluation and the $O(n\log^{2}n)$ centroid algorithm.

Table 7 reports the main comparison between canonical recipient ordering and the frozen descending shortest-transit-time order.

Descending shortest transit time tends to test rejecting recipients earlier. The smaller saving under RLNC does not imply that the ordering heuristic is less effective. In that regime, 94.14% of exact-stage candidates are retained and every correct order must evaluate all active recipients for those candidates. Complete comparisons with random, static-cut, and adjusted-cut orders are provided in Supplementary Section S2.

### 7.3. Transfer Beyond Calibrated Instances

The original calibration study asks whether useful finite-time refinement exists at prescribed static-screen strengths. Table 8 reports the within-tree calibrated results.

**Figure 6:**
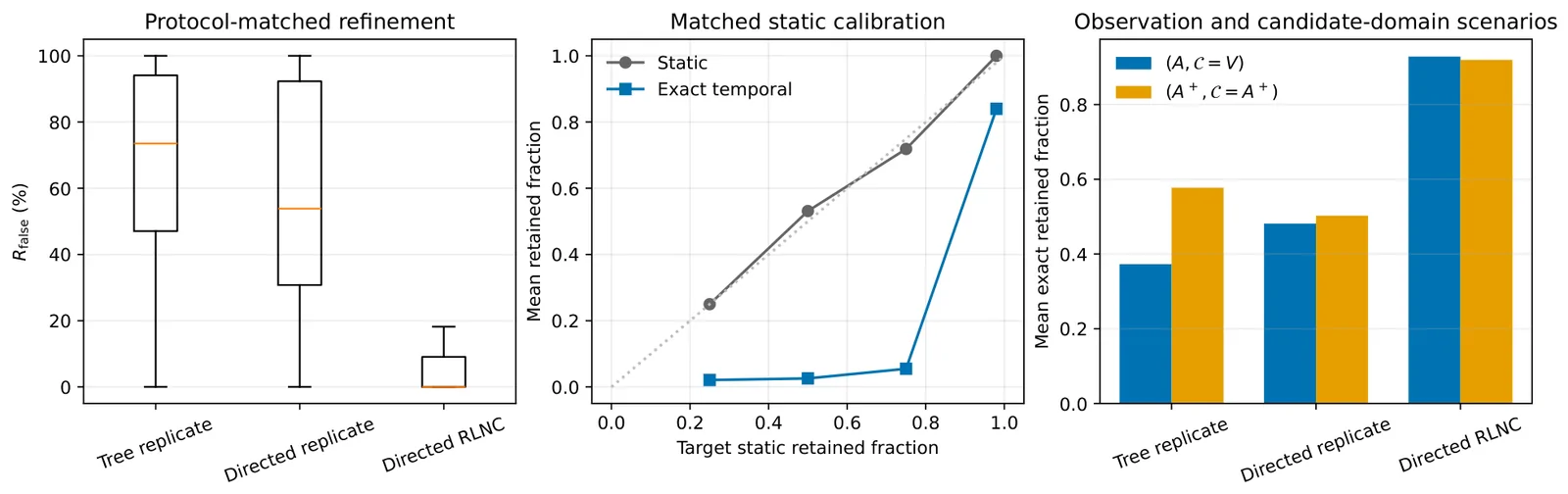
Controlled protocol refinement, matched static-screen calibration, and paired observation domains.

These results show strong conditional refinement at the three lower targets. The permissive 98% target retains every candidate under the static criterion and leaves much less room for temporal reduction. Because each configuration is selected on the same tree that is later evaluated, this study demonstrates existence and controlled sensitivity rather than transfer. Figure 6 summarizes the controlled protocol, calibration, and observation-domain comparisons.

The held-out study asks the stronger question. One static-only configuration per target is selected on 100 calibration trees and then applied unchanged to 200 unseen trees. Table 9 reports the test results.

Every one of the 800 held-out scenarios retains its true source. Test-minus-train changes in static retention range from minus 0.14 to plus 0.28 percentage points. Changes in refinement range from minus 2.69 to plus 0.13 percentage points. The three nontrivial targets therefore preserve substantial finite-time discrimination without selecting a separate configuration on each test tree. Complete frozen configurations and train and test diagnostics are provided in Supplementary Section S1.

### 7.4. Validation on Real Network Topologies

#### 7.4.1. GEANT2009 Detailed Case

GEANT2009 provides an interpretable real-network case. The scenario policy is frozen before candidate evaluation and scenario 0 is fixed before its result is known. We compare exact finite-time screening with a deadline-insensitive baseline constructed from the standard static max-flow min-cut value. The baseline retains candidate *s* when Hλ(s,a)≥K for every active recipient *a*. Across 200 decode-and-replicate scenarios, this baseline retains every candidate. Exact temporal feasibility retains 21.12% on average and 5.88% at the median. Mean relative refinement is 78.88%. Positive refinement occurs in 196 scenarios and 70 scenarios leave a singleton exact retained set. Table 10 summarizes these aggregate GEANT2009 results.

**Figure 7:**
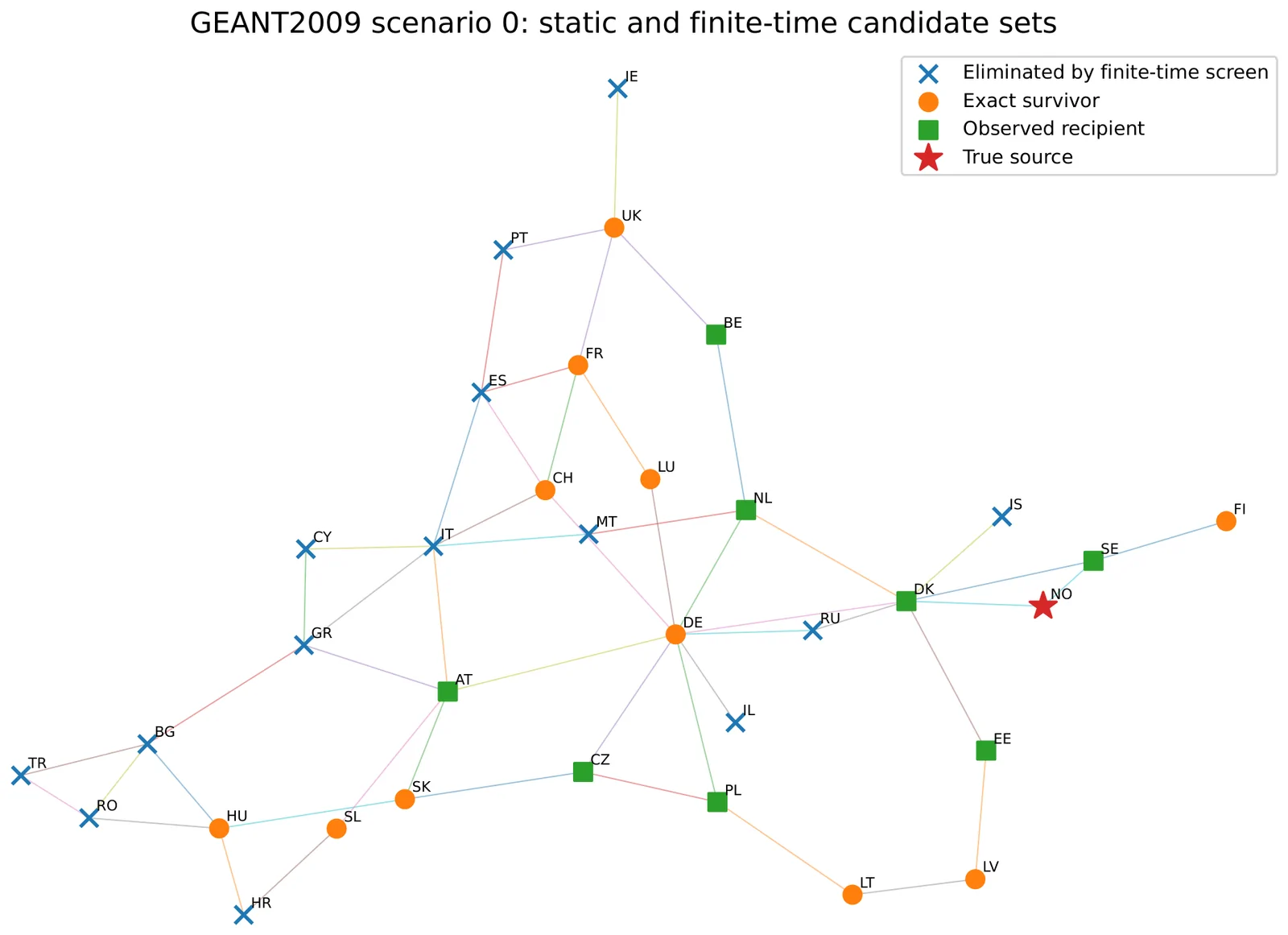
Frozen GEANT2009 scenario 0. The figure distinguishes the true source, observed recipients, candidates retained by exact temporal screening, and candidates rejected by finite-time screening.

Figure 7 shows the frozen illustrative scenario. The true source is node 29, the payload is K=32, the deadline is H=140, and the eight positive recipients are nodes 0, 1, 2, 3, 5, 23, 30, and 32. All 34 nodes pass the deadline-insensitive static max-flow min-cut baseline and 20 pass exact temporal feasibility. Fourteen candidates violate the shortest transit time. The true source remains retained. The case demonstrates a setting in which static capacity is non-discriminative and finite transport time supplies the useful information.

Complete preprocessing details, scenario-generation information, and candidate-level diagnostics are provided in Supplementary Section S3.

#### 7.4.2. Transfer Across Forty Topology Families

The broad study evaluates 40 REPETITA benchmark representations [35] derived from Internet Topology Zoo backbone families [36]. Each family contributes five frozen scenarios. The primary estimates first average the five scenarios within each family and then weight the 40 family rows equally. Table 11 shows that static feasibility remains highly permissive, while exact temporal feasibility retains about one quarter of the candidate population.

The deterministic 95% intervals for the overall means are [98.39%, 99.93%] for static retention, [18.91%, 32.41%] for exact retention, and [67.45%, 80.95%] for refinement. All 200 scenarios retain their true source, and every exact set is contained in its static set.

The suite contains 16,960 candidate evaluations. Shortest transit time rejects 11,982 candidates. Static and deadline-adjusted cuts reject 53 and 70. The exact temporal oracle rejects another 1204 among the 4855 candidates that reach it. The final retained count is 3651. The staged design therefore combines inexpensive broad screening with additional exact refinement. It uses 29,338 recipient-oracle calls, or 1.73 calls per candidate under candidate weighting and 2.03 under equal-family weighting. Complete per-network and subgroup results are provided in Supplementary Section S4.

### 7.5. Fidelity of the Real-Network Representation

The real-network result relies on controlled per-network normalization of REPETITA benchmark attributes. A separate audit therefore tests whether delay discretization preserves the shortest-path quantities used by the preliminary screen. It parses the raw delays exactly and compares every ordered pair of distinct nodes independently of source-identification outcomes.

Across 336,560 ordered pairs, the pair-weighted mean reconstructed shortest-distance error is 1.64%. The median is 0.64% and the 95th percentile is 4.20%. A total of 96.02% of pairs remain within 5% and 98.60% remain within 10%. The set of arcs belonging to at least one shortest path is unchanged for 90.66% of pairs and has a mean Jaccard similarity of 96.67%. Equal-family weighting gives similar values.

The audit also preserves visible limitations. Internode has a 15.95% mean error and one extreme pair with 6185% error, although its median error is 1.06% and its admissible arc set is unchanged for that extreme pair. Tinet has the lowest unchanged-set fraction at 40.28%. The suite is therefore interpreted as topology-transfer evidence under controlled benchmark parameters rather than validation under unmodified operational latencies. Full network-level fidelity results are provided in Supplementary Section S5.

### 7.6. Protocol Dependence and Tightness of the Certificate

The final question is why routing regimes yield strong refinement while P3 RLNC does not. The diagnostic separates static feasibility, ideal temporal coded feasibility, causal RLNC, and decode-before-forward transmission. Table 12 summarizes the main RLNC dynamics and protocol-gap diagnostics.

Static screening retains every candidate in P3. Exact temporal feasibility still retains 92.83% of candidates. Among the 1114 exact-feasible candidates, 1087 succeed in all 128 causal RLNC trials and the other 27 have empirical success probabilities between 0.95 and 1. No exact-feasible candidate falls below 0.95 and no exact-infeasible candidate succeeds. The mean success probability across the 100 base graphs is 0.999646 with interval [0.999436,0.999809]. Weak refinement therefore reflects a genuinely broad feasible region rather than widespread random-coding failure.

The comparison with decode-before-forward gives a different result. Only 701 of the 1114 exact-feasible candidates remain feasible under that restriction. The other 413 form a candidate-level gap of 37.07%. The mean gap across the 100 base graphs is 37.86% with interval [33.58%, 42.32%]. About 30.26% of non-source relay transmissions occur before full rank, and 47.94% of active recipients obtain innovative packets from multiple predecessors. These descriptive measurements are consistent with partial-information pipelining as an important mechanism.

The result identifies the applicability boundary. Routing-dominated operation and tight deadlines can make finite-time feasibility strongly selective. Coding-rich networks with ample capacity and temporal slack can make the exact retained set broad. In the latter setting, certification mainly removes impossible candidates and a downstream probabilistic or learned score must resolve the remaining ambiguity.

**Figure 8:**
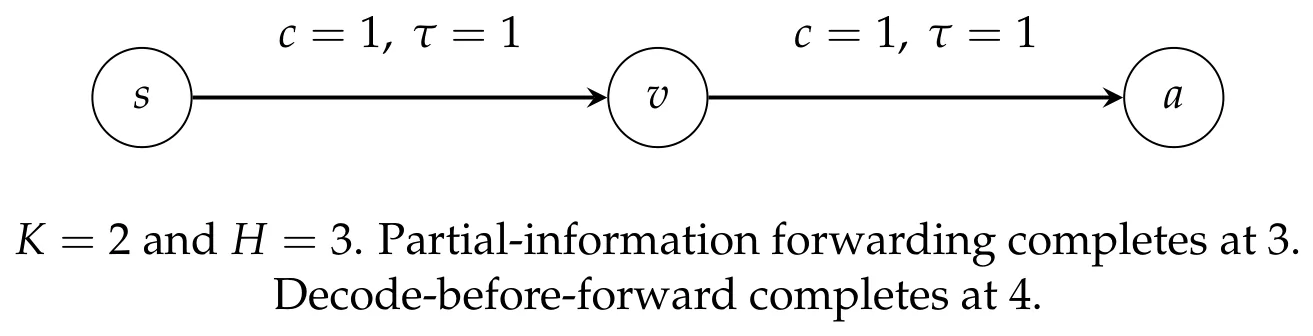
Fixed two-hop witness separating causal partial-information forwarding from decode-before-forward transmission.

Figure 8 gives the fixed two-hop witness. With unit capacities, unit transit times, payload K=2, and deadline H=3, a partially informed relay forwards the first symbol while the second remains in transit. Coded delivery completes at time 3. Decode-before-forward waits for complete relay decoding and completes at time 4.

Complete rank dynamics, structural correlations, protocol-gap categories, and bootstrap estimates are provided in Supplementary Section S6.

### 7.7. Experimental Synthesis

The experiments characterize one mathematical object from progressively stronger perspectives. Controlled protocols establish safety and show that temporal feasibility can refine static feasibility. Screening, ordering, and tree specialization make exact certification practical. Held-out calibration shows that refinement can transfer beyond the networks used for selection. GEANT2009 and the 40-family suite show that the phenomenon persists across real network topologies. The normalization audit supports the representation used for that transfer while retaining visible outliers. The RLNC diagnostic then shows that selectivity depends on how restrictive the actual transport dynamics are relative to ideal causal coding.

## 8. Discussion and Limitations

The proposed framework addresses the deliberately narrow question of whether a candidate source is incompatible with the observed recipients under specified transport constraints. It does not estimate posterior probabilities or identify the most likely source. Because causal network coding is used as an upper bound on communication capability, a retained candidate may still be infeasible under a particular uncoded protocol. By contrast, a rejected candidate cannot explain the observation under any routing or replication process that obeys the same arc capacities and transit times.

A natural extension is to use certified elimination as a preprocessing stage for an existing source-ranking method. Let $q_{\theta }\left(s|O\right)$ denote any likelihood-based, centrality-based, forensic, or learned score computed from observation O. One may first obtain the exact retained set and then select

(53) $$\hat{s}\in \arg\min_{s\in S_{H}^{\mathrm{exact}}\left(K,A,C\right)}q_{\theta }\left(s|O\right).$$

When the observation is generated by a model-consistent transmission, the assumptions of Theorem 2 hold, and the true source belongs to C, this preprocessing stage does not remove the true source. The second-stage score can then resolve ambiguity among the retained candidates. A softer alternative is to supply temporal-capacity margins or rejection-certificate features to the ranking model rather than using them as a hard restriction.

The guarantees depend on the observation and network models. The observed set contains nodes known to possess the complete message by the deadline, while the states of all other nodes remain unspecified. The framework is therefore not a complete-snapshot model and does not represent partial reception or protocol-specific decoding requirements. It also assumes fixed per-slot capacities and transit times, independent directional arcs, unlimited storage, and zero processing delay. Shared half-duplex links, wireless interference, congestion, queue-dependent delays, and time-varying networks are outside the present scope. Undirected experimental links are represented by independent antiparallel arcs and hence model full-duplex communication.

A finite upper bound on elapsed time is essential. Without a bounded deadline, any candidate that can reach all required recipients through positive-capacity paths may eventually deliver a finite message. Monotonicity provides endpoint certificates when the message size, deadline, capacities, and transit times are known only within bounded intervals, but such certificates do not replace reliable parameter measurement.

The framework is most useful as a conservative feasibility layer before probabilistic, forensic, or learned source ranking. In a communication backbone or distributed service, it can reject origins that cannot move the required payload through measured bottlenecks before an incident deadline. A prospective cybersecurity use is to test whether a suspected compromised host could have propagated a payload through a measured network before an incident deadline. In epidemic, sensor, or infrastructure settings, an analogous feasibility test is appropriate only when capacities, propagation delays, message size, and deadline have a defensible interpretation under the physical model of that application. These uses do not turn feasibility into attribution. Retained candidates must still be distinguished using domain evidence, content, timestamps, or a stochastic diffusion model.

The expanded experiments clarify several limitations. Static feasibility may be almost non-discriminative when the product of deadline and static cut greatly exceeds the payload, as in GEANT2009 and many normalized real-network scenarios. Exact retention is coded temporal feasibility, not a guarantee under every actual protocol. The RLNC diagnostic shows close operational agreement for a large field and permissive P3 regime, but decode-before-forward creates a much larger gap. The 40-family suite uses controlled relative REPETITA benchmark attributes rather than operator-measured capacities and delays, spans 50–197 nodes rather than Internet-scale graphs, and uses five scenarios per family. Its intervals therefore quantify variation across the 40 observed family means, not all possible sources, observations, or networks. Delay normalization preserves shortest paths well on average but has notable outliers.

Future work should develop protocol-aware certificates between ideal coding and restrictive routing, incorporate uncertain or time-varying capacity and congestion, and model partial reception rather than only complete-message observations. Joint systems could combine feasibility elimination with probabilistic or neural rankings, while adaptive sensor placement could select observations that maximize safe rejection. Parallel or GPU temporal-flow solvers would extend the exact stage to larger networks. Finally, deployment-level validation requires networks with measured transport attributes, message sizes, deadlines, and independently established source events.

## 9. Conclusions

This work develops a deterministic framework for ruling out source candidates when an observed transmission must be completed within a finite deadline. By modeling message size, arc capacities, transit times, observed recipients, and the deadline jointly, source identification becomes a question of deadline-constrained transport feasibility rather than likelihood ranking. The time-expanded network provides an exact causal network-coding characterization, while a minimum-cost circulation reduction enables exact pairwise computation on the original network. Monotonicity further yields endpoint certificates when the model parameters are known only within intervals.

The framework becomes especially explicit on bidirected trees. The finite-time value reduces to a path formula involving total transit time and bottleneck capacity. This leads to an intersection-of-balls characterization and linear-time all-candidate evaluation under uniform capacities, together with an $O(|V|\log^{2}|V|)$ centroid decomposition algorithm under heterogeneous capacities.

The protocol-generated experiments were consistent with the safety guarantee. The true source was retained in all 300 independent base-graph trials, while the held-out calibration retained every true source across 800 previously unseen test scenarios. Descending shortest-transit-time recipient order reduced exact temporal calls without changing any candidate decision. These results support using safe preliminary screens and early stopping before the exact oracle.

The real-network studies extend the evidence beyond small synthetic graphs. In GEANT2009, exact temporal feasibility removed 78.88% of the permissive static retained set on average. Across 40 equally weighted backbone topology families with 50–197 nodes, static and exact retention averaged 99.27% and 25.26%, respectively, giving mean relative refinement of 74.45% with zero true-source eliminations. The associated normalization audit shows good shortest-path fidelity on average while retaining visible outliers and limiting the claim to controlled benchmark transport parameters.

The RLNC diagnostic explains the negative coded-regime result. Static screening retains every P3 candidate and exact temporal feasibility remains true for 92.8%, so little refinement is available. Causal RLNC realizes every exact-feasible candidate with empirical probability at least 0.95, whereas decode-before-forward is infeasible for approximately 37% of exact-feasible candidates. The practical meaning of retention therefore depends on the permitted relay protocol. Future deployment studies should combine these certificates with source rankings and validate them on networks with measured capacities, transit times, message sizes, deadlines, and independently established source events. 

## Figures and Tables

**Table 1:** Experimental sequence and primary unit of analysis.

Study	Main Question	Population	Primary Unit
Controlled protocols	Safety and selectivity	P1, P2, and P3 with 100 graphs each	Base graph
Controlled tree factors	Capacity, delay, recipients, and uncertainty	100 paired 64-node trees	Base tree
Computation	Screening and all-candidate scaling	Directed graphs and trees up to 2048 nodes	Graph seed
Held-out transfer	Generalization beyond calibration	100 calibration trees and 200 held-out trees across four targets	Test tree
GEANT2009	Detailed real-network behavior	200 frozen scenarios on one 34-node topology	Scenario
Forty-family suite	Transfer across real network topologies	40 families with five scenarios each	Network family
Normalization audit	Fidelity of delay discretization	336,560 ordered node pairs	Network family
RLNC diagnostic	Protocol dependence of exact retention	100 graphs, 12 candidates per graph, and 128 trials per graph-candidate pair	Base graph

**Table 2:** Protocol-generated matched results. Mean static and mean exact are retained-candidate counts. Intervals are seeded 95% bootstrap intervals for mean false-candidate refinement. The final column gives false eliminations and the exact one-sided 95% upper rate bound for each protocol design [34]. DR denotes decode-and-replicate transmission.

Protocol	Scenario	Mean Static	Mean Exact	Mean $R_{\mathrm{false}}$	95% CI for Mean	Median $R_{\mathrm{false}}$	False Eliminations and 95% Upper
Tree DR	Full (A,V)	18.00	6.71	66.41%	[60.53, 72.41]%	73.53%	0/100 and 2.95%
Tree DR	Source-observed $\left(A^{+},A^{+}\right)$	9.46	5.46	47.28%	[39.62, 55.01]%	50.00%	0/100 and 2.95%
Directed DR	Full (A,V)	13.98	6.74	55.85%	[49.31, 62.23]%	53.85%	0/100 and 2.95%
Directed DR	Source-observed $\left(A^{+},A^{+}\right)$	7.91	4.00	57.12%	[50.16, 63.91]%	62.50%	0/100 and 2.95%
Directed RLNC	Full (A,V)	12.00	11.14	7.82%	[5.55, 10.36]%	0.00%	0/100 and 2.95%
Directed RLNC	Source-observed $\left(A^{+},A^{+}\right)$	11.71	10.76	8.78%	[6.31, 11.58]%	0.00%	0/100 and 2.95%

**Table 3:** Comparison of approximately mean-matched capacity and transit-time conditions for core recipients at K=160 and H=40. Retained values are mean candidate counts over 100 paired trees.

Regime	Shortest Retained	Static Retained	Exact Retained	Relative Refinement
Uniform capacity and uniform transit time	62.24	64.00	16.55	74.14%
Heterogeneous capacity and uniform transit time	62.24	46.00	8.50	81.52%
Uniform capacity and heterogeneous transit time	58.51	64.00	16.29	74.55%
Heterogeneous capacity and heterogeneous transit time	58.51	46.00	8.93	80.59%

**Table 4:** Isolated screening-stage profile over three directed graphs. Times are median $\left[Q_{1},Q_{3}\right]$ over 30 measurements. Resident set size (RSS) is the absolute process-memory measurement. Allocation peak is measured separately. Witnesses are candidate–recipient rejections out of 138 evaluations.

Stage	Time in ms	RSS in MiB	Peak Allocation in KiB	Witnesses
Shortest transit	1.508 [1.360,1.646]	108.29	17.83	0
Static cut	9.116 [8.544,9.938]	108.29	29.34	6
Deadline-adjusted cut	10.581 [9.830,11.361]	108.29	29.52	6
Exact temporal oracle	24.959 [21.838,32.342]	108.29	22.94	28

**Table 5:** Deadline scaling on 24-node directed graphs over three seeds. Each time is the pooled median $\left[Q_{1},Q_{3}\right]$ in milliseconds over 30 measurements. The ratio is the runtime of the time-expanded network divided by the runtime of the minimum-cost circulation.

*H*	Minimum-Cost Circulation in ms	Time-Expanded Network in ms	Time Ratio
8	0.177 [0.101,0.238]	0.937 [0.670,1.358]	5.3
16	0.178 [0.131,0.312]	10.937 [3.975,17.944]	61.5
32	0.197 [0.188,0.309]	90.219 [51.468,144.340]	456.8
64	0.193 [0.188,0.309]	482.592 [344.490,771.596]	2496.6

**Table 6:** All-candidate heterogeneous-tree scaling with |A|=|V|/2. Times are pooled medians $\left[Q_{1},Q_{3}\right]$ in milliseconds. Independent pairs denote direct evaluation of all candidate–recipient pairs using the closed-form tree expression.

|V|	Centroid in ms	Independent Pairwise Evaluation in ms	Speedup
64	0.521 [0.509,0.638]	0.975 [0.962,1.106]	1.9
128	1.151 [1.102,1.247]	4.024 [3.844,4.439]	3.5
256	2.586 [2.448,2.925]	17.241 [16.092,18.484]	6.7
512	5.732 [5.392,6.116]	70.096 [68.598,76.144]	12.2
1024	12.586 [12.050,12.909]	294.021 [286.508,303.801]	23.4
2048	24.733 [24.289,25.378]	1112.523 [1094.425,1123.266]	45.0

**Table 7:** Effect of ordering recipients by descending shortest transit time on exact temporal calls. The final column is the percentage of rejected candidates for which the first tested recipient provides the rejection. All tested orders return identical candidate decisions.

Regime	Canonical Calls	Descending-Transit Calls	Saving	First-Recipient Rejection
Directed decode-and-replicate	5233	4663	10.89%	80.29%
Directed RLNC	11,376	11,138	2.09%	84.06%

**Table 8:** Matched calibration of static-retention fractions. The four target rows reuse the same 100 base trees and are paired across target conditions. Every row has zero true-source eliminations.

Target $r_{\mathrm{static}}$	Achieved $r_{\mathrm{static}}$	Mean $R_{\mathrm{false}}$	Median $R_{\mathrm{false}}$	False Eliminations
25%	25.00%	97.73%	100.00%	0/100
50%	53.13%	98.03%	100.00%	0/100
75%	71.88%	94.40%	95.56%	0/100
98%	100.00%	16.25%	9.52%	0/100

**Table 9:** Held-out cross-network calibration on 200 unseen test trees per target. Here $r_{\mathrm{static}}$ and $r_{\mathrm{exact}}$ denote the static and exact retention fractions, respectively, and $R_{\mathrm{false}}$ denotes false-candidate refinement. All three quantities are reported as percentages. Intervals are deterministic bootstrap intervals for mean $R_{\mathrm{false}}$ over test trees.

Target $r_{\mathrm{static}}$	Test $r_{\mathrm{static}}$	Test $r_{\mathrm{exact}}$	Mean $R_{\mathrm{false}}$	95% CI for Mean $R_{\mathrm{false}}$
25%	24.43%	10.83%	59.53%	[55.19,63.52]%
50%	49.56%	31.05%	38.72%	[37.45,40.01]%
75%	74.83%	40.83%	47.11%	[45.14,49.07]%
98%	98.03%	95.62%	2.52%	[1.99,3.07]%

**Table 10:** Aggregate GEANT2009 results over 200 frozen scenarios. Retention and refinement are reported as percentages. Confidence intervals are deterministic scenario-level bootstrap intervals for the means.

Metric	Mean	Median	95% CI for Mean
Static retention	100.00%	100.00%	[100.00%, 100.00%]
Exact retention	21.12%	5.88%	[17.54%, 24.87%]
Relative refinement	78.88%	94.12%	[75.15%, 82.37%]

**Table 11:** Equal-network-family aggregate results for the 40-family suite. For each network, the five scenarios are first averaged, after which the network-family means are weighted equally. Static retention, exact retention, relative refinement, and exact-stage fraction are reported as percentages. The exact-stage fraction is the fraction of candidate evaluations that reach the exact temporal oracle after preliminary screening. Capacity groups are prespecified and descriptive.

Population	Networks	Static Retention	Exact Retention	Relative Refinement	Exact-Stage Fraction
All families	40	99.27%	25.26%	74.45%	28.63%
Uniform capacity	31	100.00%	17.18%	82.82%	22.31%
Heterogeneous capacity	9	96.77%	53.09%	45.63%	72.32%

**Table 12:** RLNC dynamics and protocol-gap results on the 100 frozen P3 base graphs. Each graph has 12 candidate sources and each graph-candidate pair is evaluated over 128 coding trials. Here $\hat{p}$ denotes the empirical RLNC success probability.

Quantity	Result
Candidate instances	1200
Statically feasible candidates	1200
Exact temporally feasible candidates	1114
Exact feasible with $\hat{p}=1$	1087
Exact feasible with $0.95\leq \hat{p}<1$	27
Exact feasible but decode-before-forward infeasible	413
Mean fraction of relay transmissions before full rank	30.26%
Mean fraction of recipients with multiple predecessors	47.94%

## Data Availability

The study uses synthetic graph instances and benchmark representations of real network topologies. Frozen configurations, instance-level JSON Lines and CSV files, publication-table CSV files, plotting code, environment metadata, artifact hashes, source code, and reproducibility tests are available at https://github.com/ZimengInfo/SourceCandidateCertifiedElimination (accessed on 3 September 2026). The repository additionally provides download scripts and frozen manifests for the Internet Topology Zoo and REPETITA inputs, the GEANT2009 and 40-family result artifacts, the normalization-fidelity audit, and the RLNC dynamics diagnostic. Raw third-party datasets remain distributed through their respective upstream repositories. A versioned archival release will accompany publication.

## Supplementary Materials

The following supporting information can be downloaded at https://www.mdpi.com/article/10.3390/e28091022/s1. The separately submitted Supplementary Materials contain Section S1 on common experimental definitions and held-out transfer, Section S2 on recipient ordering and early rejection, Section S3 on GEANT2009 preprocessing and the detailed case study, Section S4 on the forty-family real-network transfer study, Section S5 on delay-normalization fidelity in the forty-family suite, and Section S6 on RLNC dynamics and the protocol-feasibility gap. They include Tables S1–S14 and Figures S1–S6.

## Abbreviations

The following abbreviations are used in this manuscript:

DR	Decode-and-replicate
FER	False-elimination rate
GEANT	Pan-European research and education network topology
JML	Joint maximum likelihood
ML	Maximum likelihood
REPETITA	Repeatable traffic-engineering benchmark framework
RLNC	Random linear network coding
SI	Susceptible–Infectious

## Notations

The following notations are used in this manuscript:

*G*	Directed network
*V*	Node set of *G*
*E*	Directed arc set of *G*
*e*	Directed arc in *E*
u,v	Nodes in *V*
$c_{e}$	Per-slot transmission capacity of arc *e*
$\tau_{e}$	Transit time of arc *e*
$\delta^{+}\left(B\right)$	Outgoing directed arc boundary of node set *B*
$\delta^{-}\left(B\right)$	Incoming directed arc boundary of node set *B*
$P_{sa}$	Set of directed paths from candidate *s* to recipient *a*
$d_{\tau }\left(s,a\right)$	Shortest-transit distance from *s* to *a*
*K*	Number of independent symbols in the finite message
*H*	Common delivery deadline
$T_{H}$	Discrete time horizon {0,1,…,H}
*A*	Set of observed nodes known to possess the complete message by *H*
C	Candidate-source domain
$A_{s}$	Required recipient set A∖{s} for candidate *s*
$G_{H}$	Time-expanded network associated with deadline *H*
$M_{H}$	Storage-arc capacity used in the time-expanded network
λ(s,a)	Static minimum-cut value from candidate *s* to recipient *a*
$\lambda_{H}\left(s,a\right)$	Exact temporal minimum-cut value from *s* to *a* by deadline *H*
$\Phi_{H}\left(s;A\right)$	Minimum exact temporal value over the required recipients in $A_{s}$
ρ(s;A)	Minimum static cut over the required recipients in $A_{s}$
$D_{\tau }\left(s;A\right)$	Maximum shortest-transit distance from *s* to the required recipients
${\bar{\lambda }}_{H}\left(s,a\right)$	Deadline-adjusted cut upper bound for the pair (s,a)
${\bar{\Phi }}_{H}\left(s;A\right)$	Minimum deadline-adjusted cut value over the required recipients
$S_{H}^{\mathrm{exact}}$	Candidate set retained by exact temporal feasibility
$S_{H}^{\mathrm{static}}$	Candidate set retained by deadline-insensitive static feasibility
$S_{H}^{\mathrm{adjusted}}$	Candidate set retained by the deadline-adjusted cut screen
Θ(s;A,K)	Minimum deadline required for candidate *s* on a bidirected tree
$b_{sa}$	Minimum arc capacity along the unique tree path from *s* to *a*

## Appendix A. Proof of Theorem 1

**Proof.** Fix a candidate *s* and a deadline *H*. The time-expanded network $G_{H}$ is acyclic because every transmission or storage arc moves from time *t* to a strictly larger time. The storage capacity $M_{H}$ in (10) is larger than the sum of the capacities of all transmission arcs in $G_{H}$. Hence no minimum source–terminal cut needs to use a storage arc. Replace every integer-capacity arc of $G_{H}$ by the corresponding number of parallel unit-capacity arcs. This transformation preserves all cut values. The single-source multicast theorem states that a *K*-dimensional common message can be delivered from (s,0) to every terminal (a,H), $a\in A_{s}$, if and only if each source–terminal minimum cut is at least *K* [3]. The theorem may be implemented by a linear network code over a sufficiently large finite field [21,22]. Since $G_{H}$ is acyclic and topologically ordered by time, every local encoding operation uses only symbols arriving in earlier layers and is therefore causal. If $\Phi_{H}\left(s;A\right)\geq K$, every terminal cut is at least *K*, so the multicast theorem supplies a causal code. Conversely, suppose a causal code delivers *K* independent message symbols to terminal (a,H). For any cut separating (s,0) from (a,H), all information available at the terminal must cross the cut. The cut-set bound therefore requires cut capacity at least *K*. This holds for every $a\in A_{s}$, so $\Phi_{H}\left(s;A\right)\geq K$. The same converse applies to any routing or replication protocol. Therefore, $\Phi_{H}\left(s;A\right)<K$ is a conservative rejection certificate even when the actual process does not use coding. □

## Appendix B. Proof of Theorem 2

**Proof.** For each $a\in A_{s^{*}}$, any successful protocol must communicate *K* independent symbols across every $\left(s^{*},0\right)$–(a,H) cut of $G_{H}$. Hence $\lambda_{H}\left(s^{*},a\right)\geq K$. Taking the minimum over the required recipients gives $\Phi_{H}\left(s^{*};A\right)\geq K$, and therefore $s^{*}\in S_{H}^{\mathrm{exact}}\left(K,A,C\right)$. □

## Appendix C. Proof of Theorem 3

**Proof.** Fix a candidate *s* in the left-hand retained set of (17). By Theorem 1, there exists a causal code that delivers *K* symbols to every node in $A_{s}$ by deadline *H* under parameters (c,τ). The same transmissions remain admissible under capacities $c^{\prime }\geq c$. If an arc transit time decreases, each affected symbol arrives no later than in the original schedule and may be stored until the time at which the original code used it. Hence the code remains causal under $\tau^{\prime }\leq \tau$. The later deadline $H^{\prime }\geq H$ preserves every on-time delivery, and the smaller payload $K^{\prime }\leq K$ can be obtained by restricting the code to any $K^{\prime }$ independent source symbols. Finally, $A^{\prime }\subseteq A$ implies $A_{s}^{\prime }\subseteq A_{s}$, so the second instance imposes no additional recipient requirement. Thus, *s* belongs to the right-hand retained set. □

## Appendix D. Proof of Corollary 1

**Proof.** Every admissible realization is no more favorable than $\left(K_{-},H_{+},c^{+},\tau^{-}\right)$ and no less favorable than $\left(K_{+},H_{-},c^{-},\tau^{+}\right)$. Theorem 3 therefore gives

(A1) $$S^{\mathrm{unfav}}\subseteq S_{H}^{\mathrm{exact}}\left(K,A,C;c,\tau \right)\subseteq S^{\mathrm{fav}}$$

for every admissible (K,H,c,τ). A candidate outside $S^{\mathrm{fav}}$ is consequently rejected in every realization, and a candidate inside $S^{\mathrm{unfav}}$ is retained in every realization. Candidates in $S^{\mathrm{fav}}\setminus S^{\mathrm{unfav}}$ are not classified uniformly by the endpoint inclusions. □

## Appendix E. Static-Flow Formulation and Proof of Proposition 1

For distinct s,a∈V, let

(A2) $$\begin{array}{cc} F_{s,a}:=\{x\in R_{+}^{E}:\; & 0\leq x_{e}\leq c_{e}\;\mathrm{for}\;\mathrm{all}\;e\in E,\\ & \sum_{e\in \delta^{+}\left(v\right)}x_{e}-\sum_{e\in \delta^{-}\left(v\right)}x_{e}=0\;\mathrm{for}\;\mathrm{all}\;v\in V\setminus \left\{s,a\right\},\\ & \sum_{e\in \delta^{+}\left(s\right)}x_{e}-\sum_{e\in \delta^{-}\left(s\right)}x_{e}\geq 0\}, \end{array}$$

and define

(A3) $$|x|:=\sum_{e\in \delta^{+}\left(s\right)}x_{e}-\sum_{e\in \delta^{-}\left(s\right)}x_{e}.$$

The equivalent static-flow formulation is

(A4) $$\lambda_{H}\left(s,a\right)=\min_{x\in F_{s,a}}\left\{\left(H+1\right)|x|-\sum_{e\in E}\tau_{e}x_{e}\right\}.$$

**Proof.** Fix distinct nodes *s* and *a*. Since every original arc has positive transit time, an optimizer of (A4) may be chosen without flow-carrying directed cycles. Such a flow admits a path decomposition

(A5) $$x=\sum_{P\in P_{sa}}x_{P}\chi^{P},\;x_{P}\geq 0,\;\sum_{P\in P_{sa}}x_{P}=\left|x\right|,$$

where $P_{sa}$ is the set of directed *s*–*a* paths and $\chi^{P}$ is the incidence vector of *P*. Let

(A6) $$\tau \left(P\right):=\sum_{e\in P}\tau_{e}.$$

Repeating the path flow of value $x_{P}$ in every admissible departure slot delivers

(A7) $$x_{P}{\left[H-\tau \left(P\right)+1\right]}_{+}$$

symbols by the deadline. An optimal decomposition may set $x_{P}=0$ whenever τ(P)>H, because deleting a path with a nonpositive repetition coefficient preserves feasibility and does not decrease the objective. Therefore,

(A8) $$\begin{array}{cc} \sum_{P\in P_{sa}}x_{P}\left(H-\tau (P)+1\right)\; & =\left(H+1\right)|x|-\sum_{P\in P_{sa}}x_{P}\tau \left(P\right)\\ & =\left(H+1\right)|x|-\sum_{e\in E}\tau_{e}x_{e}. \end{array}$$

The maximum-flow-over-time theorem states that an optimal temporally repeated static flow attains the maximum amount deliverable by deadline *H* [2]. Since this amount also equals the maximum flow, and hence the minimum cut, in $G_{H}$, Equation (A4) follows. For the circulation formulation, add the return arc a→s described in Proposition 1. Closing path *P* through this return arc creates a cycle whose cost per unit is

(A9) τ(P)−(H+1).

Thus, closing the path decomposition in (A5) produces a circulation with a cost equal to the negative of (A8). Conversely, every negative-cost cycle in the augmented network contains the return arc, because all original arc costs are positive. Removing the return arc from such a cycle leaves an *s*–*a* walk, from which positive-cost directed cycles may be deleted to obtain an *s*–*a* path without increasing cost. Hence every minimum-cost circulation corresponds to a feasible combination of temporally repeated path flows. The capacity $\sum_{e\in \delta^{+}\left(s\right)}c_{e}$ on the return arc is sufficient because the value of any feasible static *s*–*a* flow is at most the total capacity leaving *s*. It follows that the minimum circulation cost is $-\lambda_{H}\left(s,a\right)$. Integral capacities imply the existence of an integral optimum. □

## Appendix F. Proof of Proposition 2

**Proof.** If $d_{\tau }\left(s,a\right)>H$, every directed *s*–*a* path has transit time larger than the deadline. Hence $G_{H}$ contains no path from (s,0) to (a,H), and $\lambda_{H}\left(s,a\right)=0$. For the static bound, let *B* attain the static minimum cut in (29). In $G_{H}$, place (v,t) on the source side for every $t\in T_{H}$ exactly when v∈B. No storage arc crosses this repeated partition. Each original cut arc $e\in \delta^{+}\left(B\right)$ contributes ${\left[H-\tau_{e}+1\right]}_{+}$ transmission copies, and $\tau_{e}\geq 1$ gives

(A10) $${\left[H-\tau_{e}+1\right]}_{+}\leq H.$$

The repeated temporal cut therefore has a capacity of at most

(A11) $$H\sum_{e\in \delta^{+}\left(B\right)}c_{e}=H\lambda \left(s,a\right).$$

Taking the minimum over temporal cuts proves (32). Now let $s\in S_{H}^{\mathrm{exact}}$. For every $a\in A_{s}$, $\lambda_{H}\left(s,a\right)\geq K>0$. The first pairwise implication yields $d_{\tau }\left(s,a\right)\leq H$, so $s\in S_{H}^{\mathrm{delay}}$. The static bound yields Hλ(s,a)≥K for every $a\in A_{s}$, and therefore Hρ(s;A)≥K. Thus $s\in S_{H}^{\mathrm{static}}$, proving (33). □

## Appendix G. Deadline-Adjusted Static Cut

**Definition** **A1** (Deadline-Adjusted Static Cut)**.** *Define*

(A12) $${\bar{c}}_{e}^{\left(H\right)}:=c_{e}{\left[H-\tau_{e}+1\right]}_{+},$$

(A13) $${\bar{\lambda }}_{H}\left(s,a\right):=\min_{\begin{array}{c} B\subseteq V:\\ s\in B,a\notin B \end{array}}\sum_{e\in \delta^{+}\left(B\right)}{\bar{c}}_{e}^{\left(H\right)},$$

*and*

(A14) $${\bar{\Phi }}_{H}\left(s;A\right):=\left\{\begin{array}{cc} \min_{a\in A_{s}}{\bar{\lambda }}_{H}\left(s,a\right), & A_{s}\neq ⌀,\\ +\infty , & A_{s}=⌀. \end{array}\right.$$

*The corresponding retained set is*

(A15) $$S_{H}^{\mathrm{adjusted}}\left(K,A,C\right):=\left\{s\in C:{\bar{\Phi }}_{H}\left(s;A\right)\geq K\right\}.$$

**Theorem** **A1** (Deadline-Adjusted Screening Hierarchy)**.** *For every distinct s,a∈V,*

(A16) $$\lambda_{H}\left(s,a\right)\leq {\bar{\lambda }}_{H}\left(s,a\right)\leq H\lambda \left(s,a\right).$$

*Consequently, the set inclusion in (34) holds.*

**Proof.** Fix a static *s*–*a* cut *B*. Repeat the same original-node partition at every time layer by placing (v,t) on the source side exactly when v∈B. Storage arcs remain within one side. Every original cut arc $e\in \delta^{+}\left(B\right)$ contributes one crossing temporal copy for each admissible departure slot, so the repeated temporal cut has capacity

(A17) $$\sum_{e\in \delta^{+}\left(B\right)}c_{e}{\left[H-\tau_{e}+1\right]}_{+}.$$

Minimizing this expression over *B* gives ${\bar{\lambda }}_{H}\left(s,a\right)$, while the unrestricted temporal minimum cut can only be smaller. Hence $\lambda_{H}\left(s,a\right)\leq {\bar{\lambda }}_{H}\left(s,a\right)$. Let $B^{*}$ attain λ(s,a). Since $\tau_{e}\geq 1$,

(A18) $${\bar{\lambda }}_{H}\left(s,a\right)\leq \sum_{e\in \delta^{+}\left(B^{*}\right)}c_{e}{\left[H-\tau_{e}+1\right]}_{+}\leq H\sum_{e\in \delta^{+}\left(B^{*}\right)}c_{e}=H\lambda \left(s,a\right).$$

Taking the minimum over $a\in A_{s}$ and applying the threshold *K* gives the set inclusions in (34). □

Neither pairwise inequality has a universal constant-factor reverse bound. For ${\bar{\lambda }}_{H}\left(s,a\right)\leq H\lambda \left(s,a\right)$, a unit-capacity arc with transit time *H* has $\lambda_{H}={\bar{\lambda }}_{H}=1$ but Hλ=H. For $\lambda_{H}\left(s,a\right)\leq {\bar{\lambda }}_{H}\left(s,a\right)$, consider a two-arc unit-capacity path with both transit times equal to *m* and deadline H=2m. Then $\lambda_{H}=1$ while ${\bar{\lambda }}_{H}=m+1$.

## Appendix H. Size of the Time-Expanded Network

The construction in Definition 3 has

(A19) $$|V_{H}\left|=\left(H+1\right)|V\right|$$

vertices and

(A20) $$|E_{H}|=H|V|+\sum_{e\in E}{\left[H-\tau_{e}+1\right]}_{+}$$

arcs.

## Appendix I. Proof of Theorem 4 and Corollary 2

**Proof.** Every static *s*–*a* flow on a bidirected tree must use the unique directed path obtained by orienting $P_{sa}$ from *s* to *a*. If its value is *f*, then

(A21) $$0\leq f\leq b_{sa},\;\sum_{e\in E}\tau_{e}x_{e}=fd_{\tau }\left(s,a\right).$$

Substitution into Proposition 1 gives

(A22) $$\begin{array}{cc} \lambda_{H}\left(s,a\right)\; & =\max_{0\leq f\leq b_{sa}}f\left(H-d_{\tau }\left(s,a\right)+1\right)\\ & =b_{sa}{\left[H-d_{\tau }\left(s,a\right)+1\right]}_{+}, \end{array}$$

which proves Theorem 4. For K>0 and $A_{s}\neq ⌀$, recipient *a* is feasible exactly when

(A23) $$b_{sa}{\left[H-d_{\tau }\left(s,a\right)+1\right]}_{+}\geq K.$$

Because $b_{sa}>0$ and the quantities are integral, this is equivalent to

(A24) $$H\geq d_{\tau }\left(s,a\right)-1+\left\lceil \frac{K}{b_{sa}}\right\rceil .$$

The smallest deadline satisfying every required recipient is therefore the maximum in (39). The cases K=0 and $A_{s}=⌀$ have no positive delivery requirement and give Θ(s;A,K)=0 by convention. □

## Appendix J. Proof of Corollary 3

**Proof.** In a connected unit-capacity tree, the unique path between every pair of distinct nodes contains only unit-capacity arcs. Its static minimum cut is therefore one, so ρ(s;A)=1 whenever $A_{s}\neq ⌀$. With unit transit times, the path quantities in Definition 8 satisfy $b_{sa}=1$ and $d_{\tau }\left(s,a\right)=d_{\mathrm{hop}}\left(s,a\right)$. Substitution into (39) yields (41). □

## Appendix K. Proof of Theorem 5

**Proof.** Common capacity makes the payload term in (39) independent of the recipient. If s∉A, then $A_{s}=A$. If s∈A, removing *s* does not change the maximum distance because the omitted distance is zero and, since |A|≥2, at least one other observed recipient remains. Therefore Θ(s;A,K)≤H is equivalent to $d_{\tau }\left(s,a\right)\leq r_{H}$ for every a∈A. Intersecting these conditions over the observed recipients and then with the candidate domain C gives (44). □

## Appendix L. Terminal-Diameter Reduction

**Lemma** **A1** (Observed-Diameter Endpoints)**.** *Let p,q∈A be endpoints of a diameter of A under $d_{\tau }$. Then, for every v∈V,*

(A25) $$\max_{a\in A}d_{\tau }\left(v,a\right)=\max\left\{d_{\tau }\left(v,p\right),d_{\tau }\left(v,q\right)\right\}.$$

**Proof.** Let *P* be the *p*–*q* path and let $L=d_{\tau }\left(p,q\right)$. For any a∈A, let *x* be the unique node where the path from *a* first meets *P*, and write $h=d_{\tau }\left(a,x\right)$. Since p,q form a diameter of *A*,

(A26) $$h+d_{\tau }\left(x,p\right)=d_{\tau }\left(a,p\right)\leq L,\;h+d_{\tau }\left(x,q\right)=d_{\tau }\left(a,q\right)\leq L.$$

Because $d_{\tau }\left(x,p\right)+d_{\tau }\left(x,q\right)=L$, these inequalities imply

(A27) $$h\leq \min\{d_{\tau }\left(x,p\right),d_{\tau }\left(x,q\right)\}.$$

Now let *y* be the node where the path from *v* first meets *P*. If *x* lies on the *y*–*q* side of *P*, then (A27) gives

(A28) $$d_{\tau }\left(y,x\right)+h\leq d_{\tau }\left(y,q\right).$$

If *x* lies on the *p*–*y* side, the symmetric inequality gives

(A29) $$d_{\tau }\left(y,x\right)+h\leq d_{\tau }\left(y,p\right).$$

Therefore

(A30) $$d_{\tau }\left(v,a\right)=d_{\tau }\left(v,y\right)+d_{\tau }\left(y,x\right)+h\leq \max\left\{d_{\tau }\left(v,p\right),d_{\tau }\left(v,q\right)\right\}.$$

Taking the maximum over a∈A proves one inequality in (A25), and the reverse inequality follows because p,q∈A. □

## Appendix M. Centroid Decomposition and Proof of Theorem 6

**Definition** **A2** (Centroid Decomposition)**.** *A centroid of a tree component is a node whose removal leaves components containing at most half of its nodes. A centroid decomposition removes a centroid and recursively decomposes the remaining components. Its depth is O(log|V|).*

**Proof.** The case K=0 is immediate, so assume K>0. For a candidate *v* with $A_{v}=⌀$, the required value is Θ(v;A,K)=0. We prove that every other candidate–recipient contribution is included exactly once and that Algorithm 2 returns their maximum. □

### Appendix M.1. First Separating Centroid

Consider a component *U* in the centroid decomposition and let *z* be its centroid. For u∈U, define

(A31) $$\ell_{z}\left(u\right):=d_{\tau }\left(z,u\right),\;\beta_{z}\left(u\right):=\min_{e\in P_{zu}}c_{e},$$

with $\ell_{z}\left(z\right)=0$ and $\beta_{z}\left(z\right)=+\infty$. Give *z* a special branch label, and for u≠z, let $\gamma_{z}\left(u\right)$ identify the component of U−z containing *u*.

**Lemma** **A2** (Unique first separation)**.** *Every ordered pair of distinct nodes (v,a) is assigned to exactly one centroid-decomposition component: the first component whose centroid z satisfies $\gamma_{z}\left(v\right)\neq \gamma_{z}\left(a\right)$.*

**Proof.** At a component containing both endpoints, either their branch labels differ, or they lie in one common component of U−z. In the latter case, the recursive call on that component is the unique child containing both endpoints. Along this recursion, the next component has at most half as many nodes, so eventually one endpoint is the centroid or the endpoints lie in different branches. This gives the existence of a first separating centroid. Before that level, the labels agree, and the pair is excluded from the through-centroid query. After separation, no recursive child contains both endpoints, so the pair cannot be assigned again. □

At the first separating centroid, the unique path passes through *z*, and therefore,

(A32) $$d_{\tau }\left(v,a\right)=\ell_{z}\left(v\right)+\ell_{z}\left(a\right),\;b_{va}=\min\left\{\beta_{z}\left(v\right),\beta_{z}\left(a\right)\right\}.$$

Pairs whose labels agree are not discarded: by Lemma A2, they are deferred to the unique recursive child containing both endpoints.

### Appendix M.2. Branch-Excluding Query

Fix v∈U and write $b_{v}:=\beta_{z}\left(v\right)$. Let $A_{U}:=A\cap U$. For an observed recipient $a\in A_{U}$ with $\gamma_{z}\left(a\right)\neq \gamma_{z}\left(v\right)$, its contribution to (39) is

(A33) $$\ell_{z}\left(v\right)-1+\ell_{z}\left(a\right)+\left\lceil \frac{K}{\min\{b_{v},\beta_{z}\left(a\right)\}}\right\rceil .$$

Partition the admissible recipients into the strict prefix $\beta_{z}\left(a\right)<b_{v}$ and the suffix $\beta_{z}\left(a\right)\geq b_{v}$. Define

(A34) $$P_{z}\left(a\right):=\ell_{z}\left(a\right)+\frac{K}{\beta_{z}\left(a\right)}\}.$$

Then the largest contribution assigned to *z* is

(A35) $$\begin{array}{cc} Q_{z}\left(v\right):=\ell_{z}\left(v\right)-1+\max\{ & \max_{\begin{array}{c} a\in A_{U}:\;\gamma_{z}\left(a\right)\neq \gamma_{z}\left(v\right)\\ \beta_{z}\left(a\right)<b_{v} \end{array}}P_{z}\left(a\right),\\ & \left\lceil \frac{K}{b_{v}}\right\rceil +\max_{\begin{array}{c} a\in A_{U}:\;\gamma_{z}\left(a\right)\neq \gamma_{z}\left(v\right)\\ \beta_{z}\left(a\right)\geq b_{v} \end{array}}\ell_{z}\left(a\right)\}, \end{array}$$

where the maximum of an empty class is −∞.

**Lemma** **A3** (Two branch maxima suffice)**.** *For branch-labeled scalar values, let $M_{g}$ be the largest value carrying branch label g. The largest value whose label differs from a queried label $g_{0}$ is determined by the two largest values among the distinct-branch maxima $\left\{M_{g}\right\}$.*

**Proof.** If the largest distinct-branch maximum does not carry label $g_{0}$, it is admissible and optimal. If it carries label $g_{0}$, the second-largest distinct-branch maximum is the largest admissible value. Only one label is excluded, so no third value is required. □

Sort $A_{U}$ by nondecreasing $\beta_{z}\left(a\right)$. Prefix summaries store the two largest distinct-branch values of $P_{z}\left(a\right)$, while suffix summaries store the two largest distinct-branch values of $\ell_{z}\left(a\right)$. A lower-bound search for $b_{v}$ places all ties $\beta_{z}\left(a\right)=b_{v}$ in the suffix, as required by the non-strict condition in (A35). Lemma A3 then returns the best value excluding branch $\gamma_{z}\left(v\right)$ in each class. Empty classes contribute −∞.

Candidate self-exclusion follows from the same branch rule. If a=v≠z, the candidate and recipient occurrences have the same branch label and are deferred together. When *v* eventually becomes a centroid, the candidate and its self-entry both carry the centroid’s special label and are excluded. Conversely, an observed centroid is available as a recipient for a candidate in any ordinary branch.

Algorithm 2 updates the value of *v* with $Q_{z}\left(v\right)$ at every centroid component containing *v*. By Lemma A2, each pair (v,a) with $a\in A_{v}$ contributes exactly one such update, and no self-pair contributes. The maximum of the updates is therefore exactly Θ(v;A,K).

### Appendix M.3. Complexity

Consider a component *U* of size *k* containing t=|A∩U| observed recipients. Finding its centroid and traversing it to compute $\ell_{z}$, $\beta_{z}$, and $\gamma_{z}$ cost O(k) time. Sorting the recipients costs O(tlogt), and constructing the prefix and suffix summaries costs O(t). Each of the *k* candidates performs one lower-bound search and constant-time branch-summary queries, for O(klog(t+1)) time. Thus the component cost is O(klogk).

At any fixed decomposition depth, the components are disjoint, and their sizes sum to at most |V|. Since logk≤log|V|, the work at one depth is O(|V|log|V|). The decomposition depth is O(log|V|), yielding total time $O(|V|\log^{2}|V|)$.

The adjacency representation, removal flags, and output array use O(|V|) space. The centroid-finding arrays and the query arrays for one component use O(k) space and are released before recursive child components are processed sequentially. The recursion stack has depth O(log|V|). Hence the live working space is O(|V|).
