# Supplementary material for: Certified Elimination of Source Candidates Under Capacity, Transit-Time, and Deadline Constraints
Source: Entropy (Basel). 2026 Sep 14;28(9):1022. doi: 10.3390/e28091022 (PMC13605446; doi:10.3390/e28091022)
Supplement: Supplementary file 1 [file entropy-28-01022-s001.zip › Definitions/logo-mdpi-eps-converted-to.pdf]

MDPI
