# Supplementary material for: Certified Elimination of Source Candidates Under Capacity, Transit-Time, and Deadline Constraints
Source: Entropy (Basel). 2026 Sep 14;28(9):1022. doi: 10.3390/e28091022 (PMC13605446; doi:10.3390/e28091022)
Supplement: Supplementary file 1 [file entropy-28-01022-s001.zip › Figures/Supplementary/appendix_delay_fidelity.pdf]

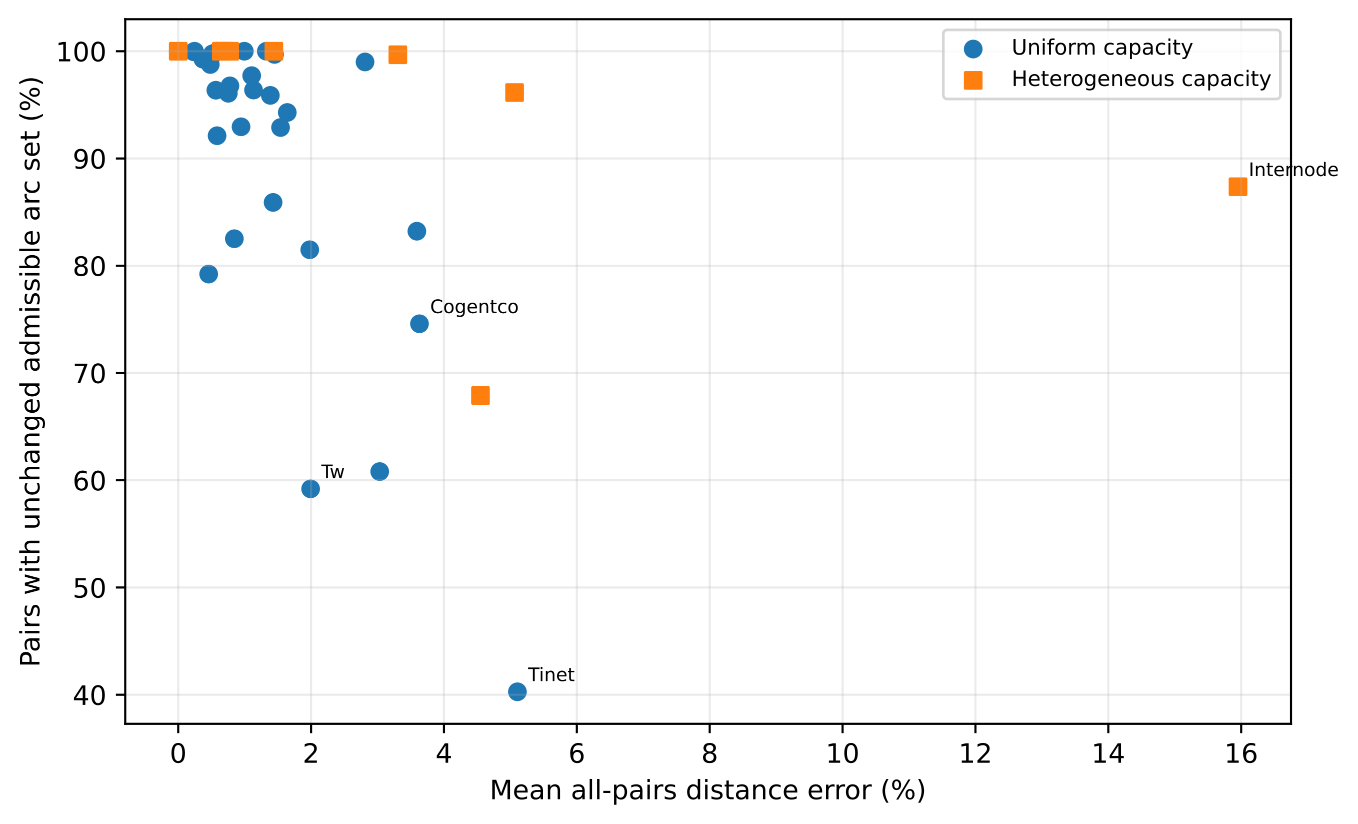

Uniform capacity
Heterogeneous capacity
Internode
Cogentco
Tw
Tinet
Pairs with unchanged admissible arc set (%)
Mean all-pairs distance error (%)
100
90
80
70
60
50
40
0
2
4
6
8
10
12
14
16
