# Supplementary material for: Certified Elimination of Source Candidates Under Capacity, Transit-Time, and Deadline Constraints
Source: Entropy (Basel). 2026 Sep 14;28(9):1022. doi: 10.3390/e28091022 (PMC13605446; doi:10.3390/e28091022)
Supplement: Supplementary file 1 [file entropy-28-01022-s001.zip › Figures/Supplementary/appendix_geant_scenarios.pdf]

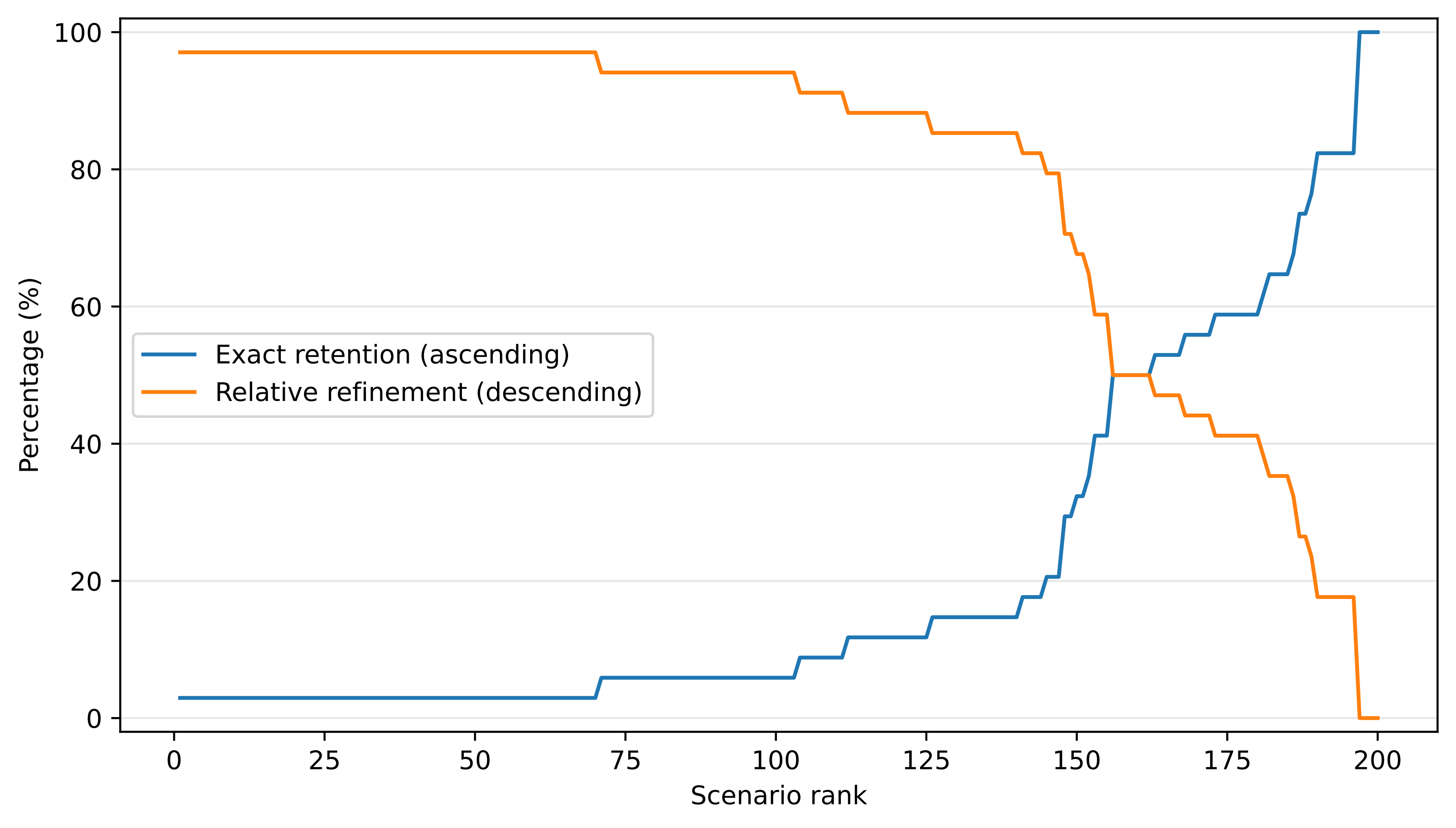
Exact retention (ascending)
Relative refinement (descending)
Percentage (%)
Scenario rank
0
20
40
60
80
100
0
25
50
75
100
125
150
175
200
