# Supplementary material for: Certified Elimination of Source Candidates Under Capacity, Transit-Time, and Deadline Constraints
Source: Entropy (Basel). 2026 Sep 14;28(9):1022. doi: 10.3390/e28091022 (PMC13605446; doi:10.3390/e28091022)
Supplement: Supplementary file 1 [file entropy-28-01022-s001.zip › Figures/Supplementary/appendix_heldout_calibration.pdf]

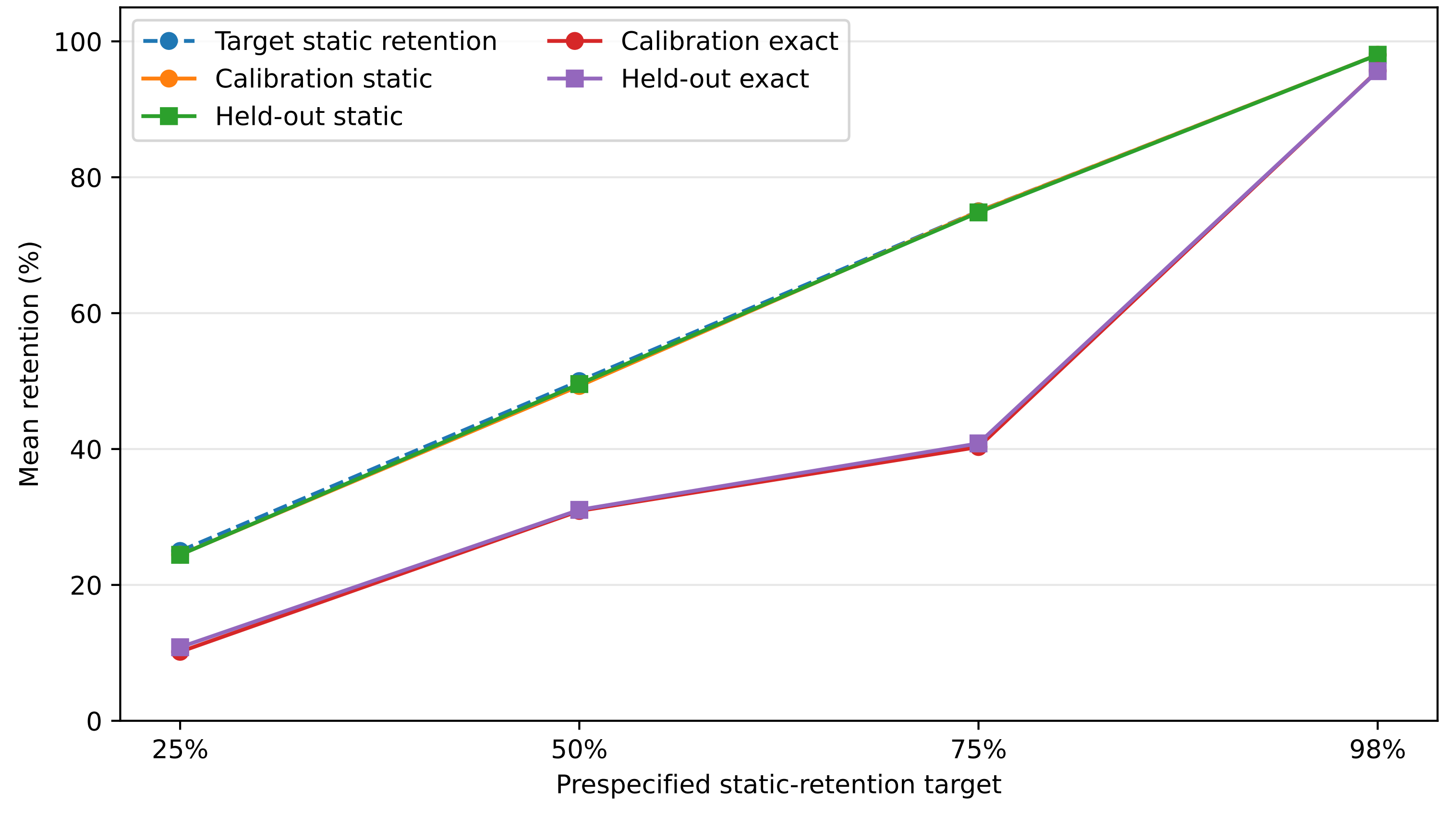

Target static retention
Calibration static
Held-out static
Calibration exact
Held-out exact
Mean retention (%)
0
20
40
60
80
100
25%
50%
75%
98%
Prespecified static-retention target
