# Supplementary material for: Certified Elimination of Source Candidates Under Capacity, Transit-Time, and Deadline Constraints
Source: Entropy (Basel). 2026 Sep 14;28(9):1022. doi: 10.3390/e28091022 (PMC13605446; doi:10.3390/e28091022)
Supplement: Supplementary file 1 [file entropy-28-01022-s001.zip › Figures/Supplementary/appendix_real_topology_transfer.pdf]

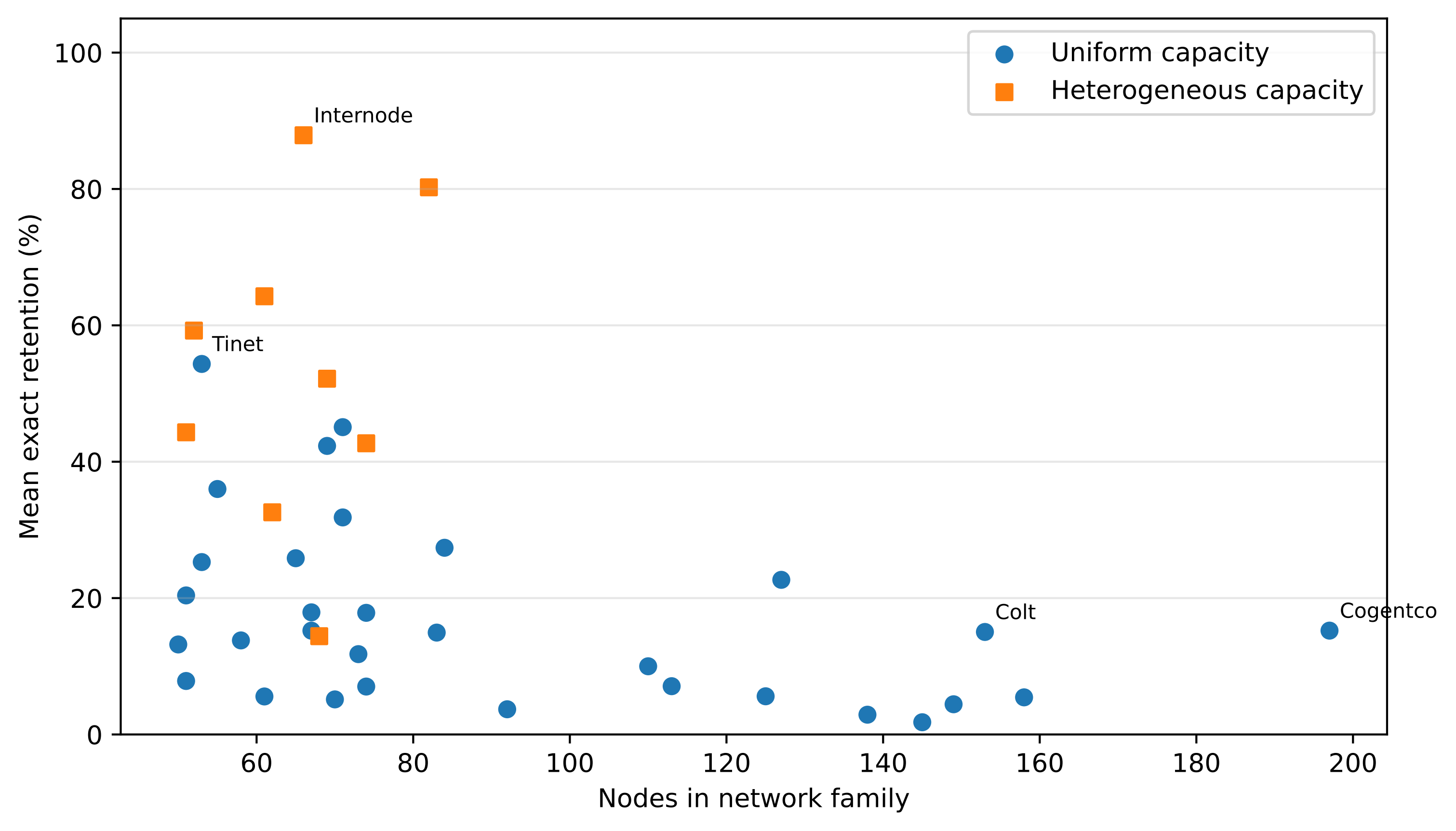
Uniform capacity
Heterogeneous capacity
Internode
Tinet
Colt
Cogentco
Mean exact retention (%)
Nodes in network family
0
20
40
60
80
100
60
80
100
120
140
160
180
200
