# Supplementary material for: Certified Elimination of Source Candidates Under Capacity, Transit-Time, and Deadline Constraints
Source: Entropy (Basel). 2026 Sep 14;28(9):1022. doi: 10.3390/e28091022 (PMC13605446; doi:10.3390/e28091022)
Supplement: Supplementary file 1 [file entropy-28-01022-s001.zip › Figures/Supplementary/appendix_recipient_ordering.pdf]

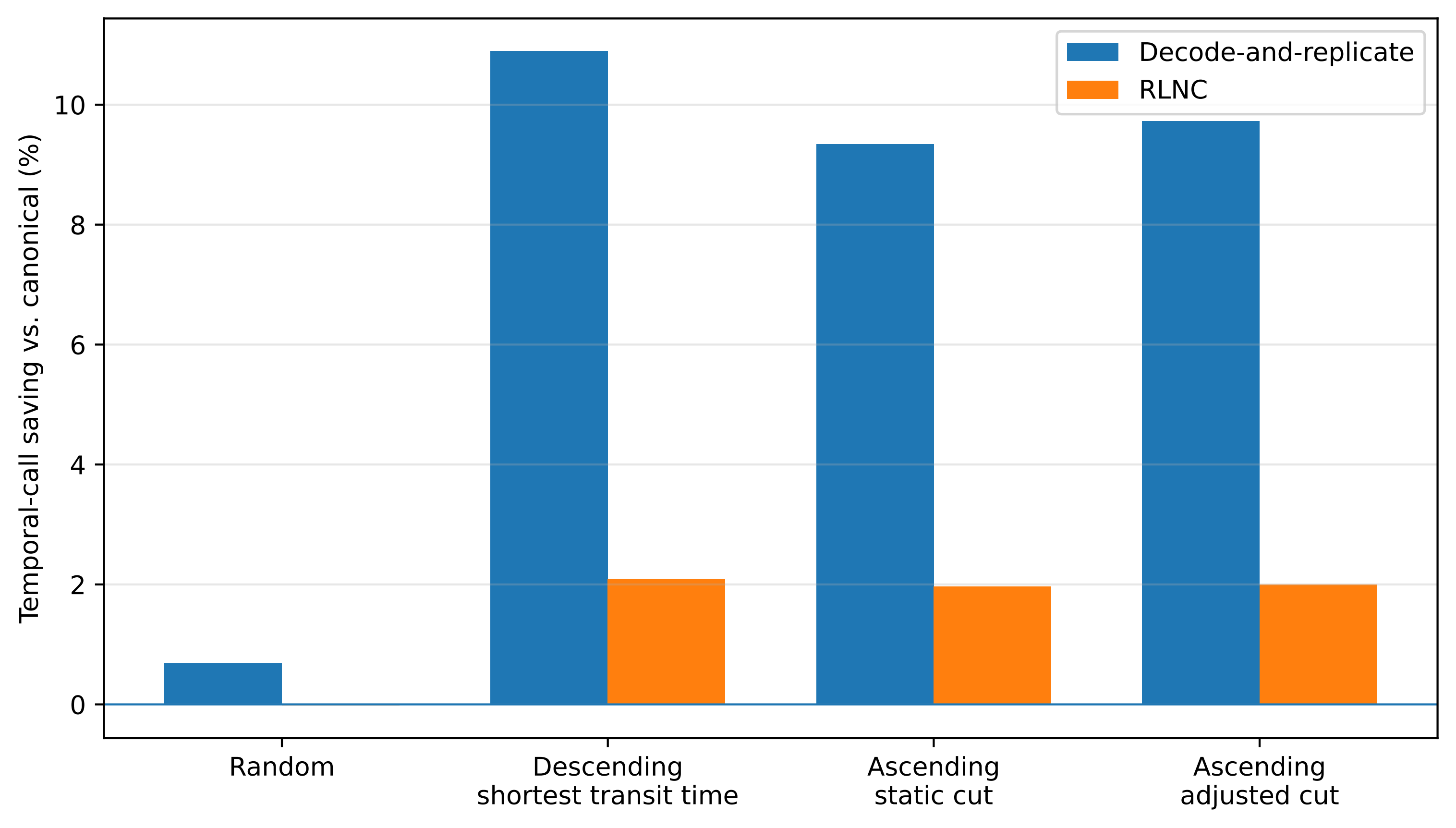

Decode-and-replicate
RLNC
Temporal-call saving vs. canonical (%)
0
2
4
6
8
10
Random
Descending
shortest transit time
Ascending
static cut
Ascending
adjusted cut
