# Supplementary material for: Certified Elimination of Source Candidates Under Capacity, Transit-Time, and Deadline Constraints
Source: Entropy (Basel). 2026 Sep 14;28(9):1022. doi: 10.3390/e28091022 (PMC13605446; doi:10.3390/e28091022)
Supplement: Supplementary file 1 [file entropy-28-01022-s001.zip › Figures/Supplementary/appendix_rlnc_protocol_gap.pdf]

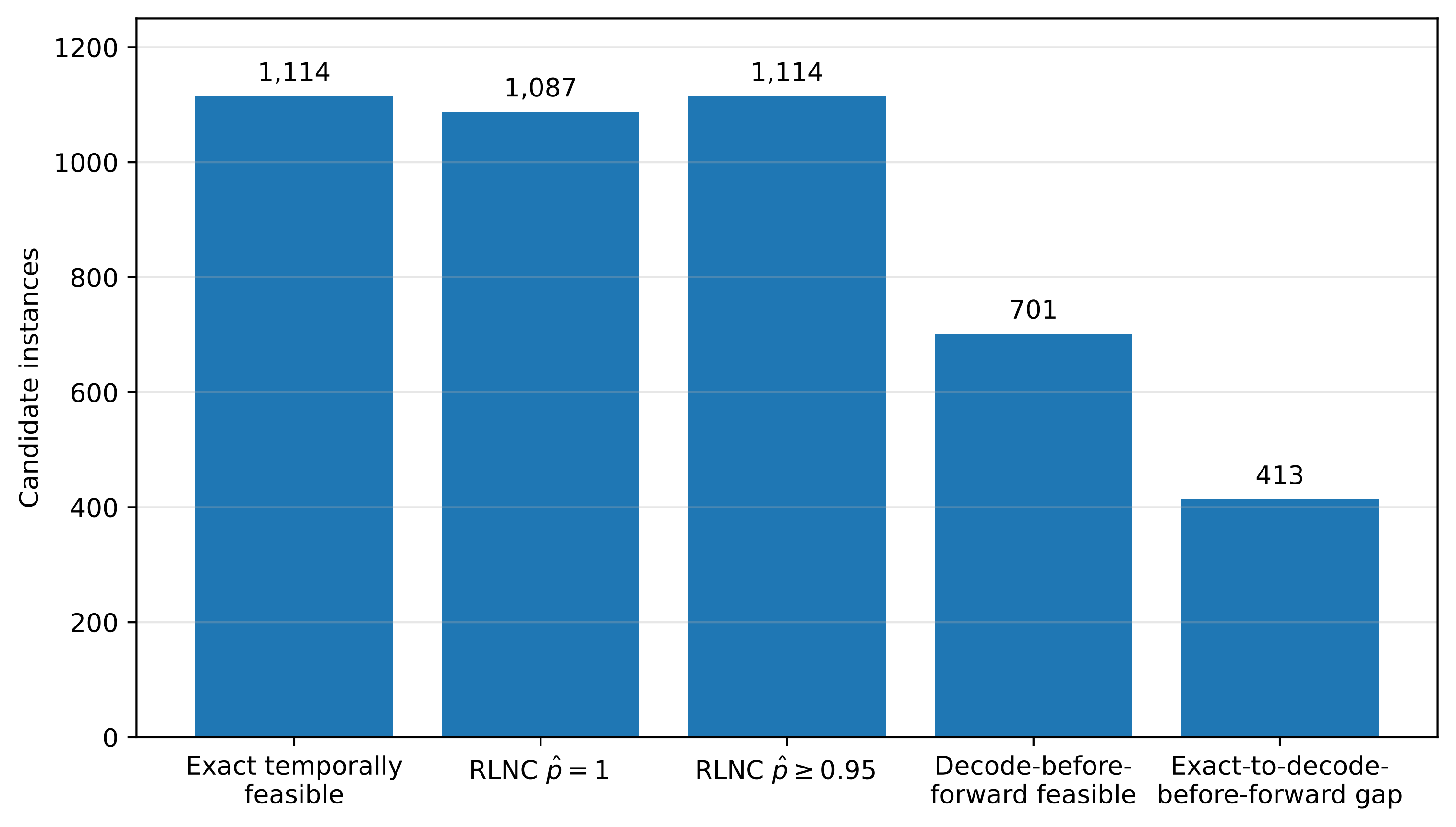

Candidate instances
1200
1000
800
600
400
200
0
1,114
1,087
1,114
701
413
Exact temporally feasible
RLNC $\hat{p} = 1$
RLNC $\hat{p} \geq 0.95$
Decode-before-forward feasible
Exact-to-decode-before-forward gap
