# Supplementary material for: Certified Elimination of Source Candidates Under Capacity, Transit-Time, and Deadline Constraints
Source: Entropy (Basel). 2026 Sep 14;28(9):1022. doi: 10.3390/e28091022 (PMC13605446; doi:10.3390/e28091022)
Supplement: Supplementary file 1 [file entropy-28-01022-s001.zip › supplementary.pdf]

Supplementary Materials: Certified Elimination of Source Candidates under Capacity, Transit-Time, and Deadline Constraints

Zimeng Wang [1], Chao Zhao [1] and Chung Chan [1,*]

S1. Common Experimental Definitions and Held-Out Transfer

For convenience, the experimental measures used in the main article are restated here. For candidate s, let $A_s = A \setminus \{s\}$ denote the active recipient set. The static and exact retention fractions are

$$r_{\text{static}} = \frac{|\mathcal{S}_H^{\text{static}}|}{|\mathcal{C}|}, \qquad r_{\text{exact}} = \frac{|\mathcal{S}_H^{\text{exact}}|}{|\mathcal{C}|}. \tag{S1}$$

When the static set is nonempty, its relative refinement is

$$R_{\text{set}} = \frac{|\mathcal{S}_H^{\text{static}}| - |\mathcal{S}_H^{\text{exact}}|}{|\mathcal{S}_H^{\text{static}}|}. \tag{S2}$$

When a true source is known, the false-elimination rate and false-candidate refinement are

$$\text{FER} = \frac{1}{N}\sum_{i=1}^{N} \mathbf{1}\Big\{ s_i^* \notin \mathcal{S}_{H,i}^{\text{exact}} \Big\}, \tag{S3}$$

and

$$R_{\text{false}} = \frac{|\mathcal{S}_H^{\text{static}} \setminus \{s^*\}| - |\mathcal{S}_H^{\text{exact}} \setminus \{s^*\}|}{|\mathcal{S}_H^{\text{static}} \setminus \{s^*\}|}. \tag{S4}$$

A zero denominator makes the corresponding refinement measure inapplicable. The deadline-insensitive static baseline is constructed from the standard static max-flow/min-cut value and retains s when $H\,\lambda(s,a) \geq K$ for every $a \in A_s$. Exact retention requires temporal coded capacity of at least K by H for every active recipient. Observations are positive-only unless stated otherwise. Nodes outside A have no asserted negative state.

The supplementary sections follow the experimental progression of the Experimental Results section in the main article. They provide the complete secondary evidence behind held-out transfer, recipient ordering, real-network evaluation, normalization fidelity, and protocol dependence.

S1.1. Held-Out Cross-Network Calibration

The original matched calibration chooses parameters separately on each tree. The transfer study instead selects one global static-only configuration for each target by using 100 calibration trees. It then evaluates the frozen configuration on 200 topologically disjoint held-out trees. The selector searches 108 configurations and never calls the temporal oracle. Calibration intervals are post-selection diagnostics. Held-out intervals provide the transfer evidence.

Table S1. Frozen global configurations selected from the calibration population.

Target r_{static}	K	Slack	Location
25%	2,560	0.00	core
50%	640	0.25	core
75%	160	0.50	core
98%	160	1.00	peripheral

Table S2. Calibration and held-out results for the frozen global configurations. Here r_{static} and r_{exact} denote static and exact retention fractions, respectively, and R_{false} denotes false-candidate refinement. These quantities are reported as percentages. CI denotes the deterministic percentile-bootstrap 95% interval for mean R_{false}.

Target r_{static}	Split	r_{static}	r_{exact}	Mean R_{false}	Median R_{false}	95% CI for mean R_{false}
25%	Train	24.45%	10.16%	62.22%	73.33%	[55.89,68.45]%
25%	Test	24.43%	10.83%	59.53%	65.15%	[55.19,63.52]%
50%	Train	49.28%	30.89%	38.59%	40.07%	[36.65,40.48]%
50%	Test	49.56%	31.05%	38.72%	39.39%	[37.45,40.01]%
75%	Train	74.97%	40.30%	47.99%	48.95%	[45.08,51.06]%
75%	Test	74.83%	40.83%	47.11%	48.89%	[45.14,49.07]%
98%	Train	98.03%	95.67%	2.51%	0.00%	[1.69,3.39]%
98%	Test	98.03%	95.62%	2.52%	0.00%	[1.99,3.07]%

Across all 1,200 calibration and test tree-target instances, the true source was never eliminated. Every exact retained set was contained in its static retained set and the heterogeneous-tree formula agreed with direct evaluation. Test-minus-train static-retention differences ranged from -0.14 to $+0.28$ percentage points. Refinement differences ranged from -2.69 to $+0.13$ percentage points.

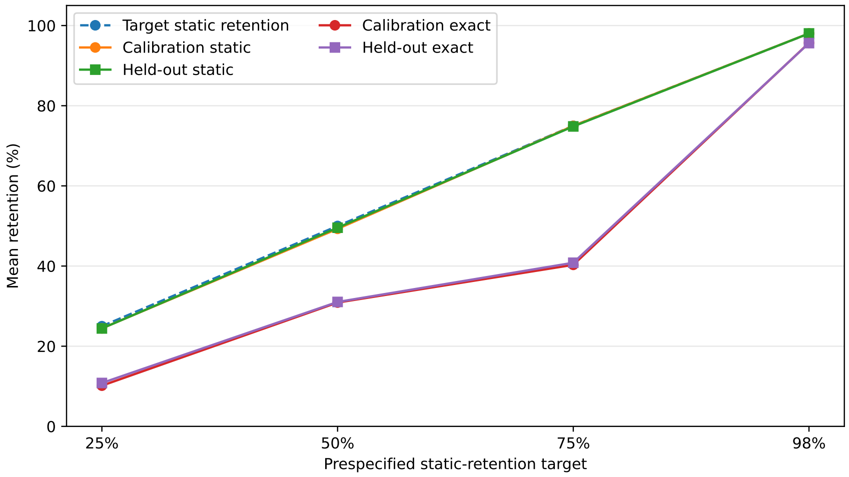

Figure S1. Calibration-to-test transfer for the four globally selected configurations. Static retention tracks the target on the held-out population, while exact retention remains lower except at the deliberately permissive 98% target.

S2. Recipient Ordering and Early Rejection

Ordering cannot change a candidate decision because feasibility requires all active recipients to pass. It can only determine how quickly a rejecting recipient is encountered. Five deterministic or seeded orders were compared on the same frozen candidate populations. The practical heuristic `delay_desc` sorts recipients by descending shortest transit time and uses quantities already computed by the preliminary screen.

Table S3. Recipient-ordering results. Saving is relative to canonical order. Bracketed values are deterministic 95% bootstrap intervals for mean network-level saving. First and first-two rates are evaluated among exact-stage rejected candidates.

**Regime**	**Ordering**	**Calls**	**Saving and 95% CI**	**Median normalized rank**	**First**	**First two**	**W/T/L**
Directed decode-and-replicate	canonical	5,233	0.00% [0.00,0.00]%	0.400	28.82%	48.82%	0/100/0
Directed decode-and-replicate	random	5,197	0.69% [-1.84,3.20]%	0.400	27.06%	50.00%	44/21/35
Directed decode-and-replicate	descending shortest transit time	4,663	10.89% [10.50,15.62]%	0.167	80.29%	92.94%	78/19/3
Directed decode-and-replicate	ascending static cut	4,744	9.34% [8.29,13.37]%	0.183	67.35%	86.18%	73/17/10
Directed decode-and-replicate	ascending adjusted cut	4,724	9.73% [8.65,13.98]%	0.167	70.29%	86.18%	74/19/7
Directed RLNC	canonical	11,376	0.00% [0.00,0.00]%	0.500	10.14%	27.54%	0/100/0
Directed RLNC	random	11,378	-0.02% [-0.67,0.56]%	0.500	13.04%	26.09%	17/61/22
Directed RLNC	descending shortest transit time	11,138	2.09% [1.50,3.06]%	0.100	84.06%	97.10%	36/64/0
Directed RLNC	ascending static cut	11,152	1.97% [1.41,2.91]%	0.100	78.26%	89.86%	35/65/0
Directed RLNC	ascending adjusted cut	11,149	2.00% [1.42,2.91]%	0.100	78.26%	91.30%	35/65/0

The descending shortest-transit-time heuristic reduced temporal calls by 10.89% in directed decode-and-replicate and 2.09% in directed RLNC. It captured 83.95% and 94.82% of the available gap between canonical order and the ideal rejecting-recipient order. The smaller saving under RLNC reflects limited avoidable work. A total of 94.14% of exact-stage RLNC candidates were retained and therefore required evaluation of every active recipient under every correct order.

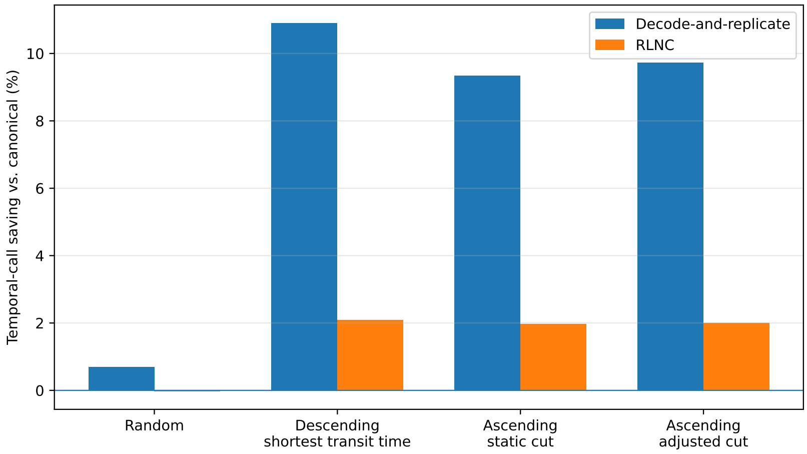

Figure S2. Temporal-oracle call savings relative to canonical recipient order. All five orders produced identical retained sets.

S3. GEANT2009 Preprocessing and Detailed Case Study

GEANT2009 is used as the detailed real-network case. The physical topology and nominal link-rate metadata are taken from the Internet Topology Zoo [1]. Benchmark delays are matched to the REPETITA representation [2]. The mapping contains 34 nodes, 52 physical links, and 104 symmetric directed arcs, with no self-loops or parallel arcs. All 52 physical links have positive original `LinkSpeedRaw` values.

S3.1. Frozen Attribute Mapping

Nominal link rates are discretized by $c_e = \max\{1, \lfloor B_e/50 + 0.5 \rfloor\}$, where B_e is measured in Mbit/s. Benchmark delays are discretized by $\tau_e = \max\{1, \lfloor d_e/25 + 0.5 \rfloor\}$. REPETITA documents latency as typically expressed in microseconds, but the experiment conservatively treats d_e as a native benchmark delay value rather than asserting an unconditional physical unit for this file.

Table S4. GEANT2009 nominal-rate mapping and directed-arc frequencies.

Nominal rate (Mbit/s)	Physical links	c_e	Directed arcs
45	2	1	4
155	2	3	4
310	1	6	2
2,500	6	50	12
10,000	41	200	82

The resulting transit times range from 11 to 194 over 42 distinct levels. Before any source experiment was run, an all-pairs structural audit found mean shortest-distance error 0.47%, p95 error 1.19%, maximum error 2.60%, and unchanged shortest-path admissible arc sets for 93.76% of ordered pairs.

S3.2. Frozen Scenario Policy and Aggregate Results

The scenario population contains 200 deterministic decode-and-replicate realizations. Sources are pseudorandomly drawn over all 34 nodes using master seed 20260901, $K = 32$, and H equal to the completion time of the tenth non-source node. Eight recipients are sampled uniformly without replacement from the non-source nodes completed by H. Scenario 0 was fixed as the illustrative case before candidate evaluation.

Table S5. GEANT2009 generation and evaluation summary.

Quantity	Minimum	Median	Mean	Maximum
Deadline H	50	81.00	100.77	244
Eligible recipients	10	10.00	10.14	11
Graph-wide completion time	200	257.50	274.40	394
Static retention	100.00%	100.00%	100.00%	100.00%
Exact retention	2.94%	5.88%	21.12%	100.00%
Relative refinement	0.00%	94.12%	78.88%	97.06%

Positive refinement occurred in 196 of 200 scenarios and exact screening produced a singleton retained set in 70 scenarios. Across 6,800 candidate evaluations, 1,442 candidates reached the exact stage and 10,931 recipient-level temporal calls were made. The deadline-insensitive static max-flow/min-cut baseline retained every candidate in every scenario. Under the frozen GEANT operating regime, static capacity feasibility is therefore non-discriminative, while finite transit times and deadlines provide the selectivity. No true source was eliminated and every exact retained set was contained in its static retained set.

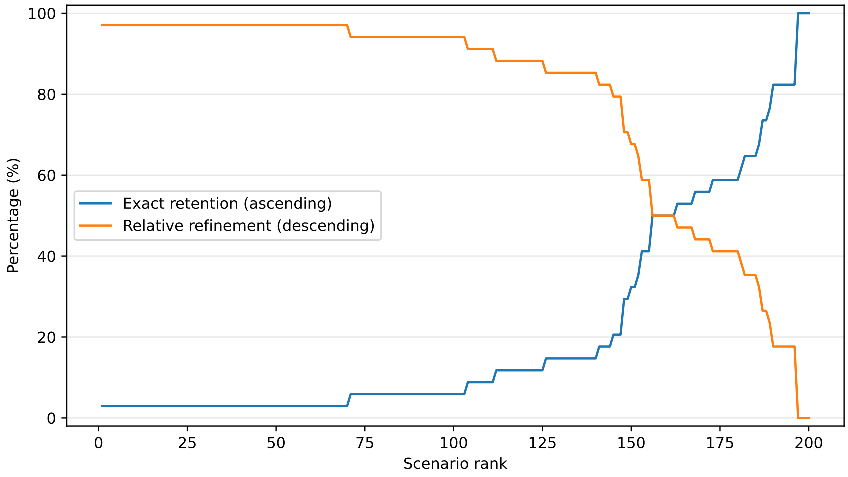

Figure S3. Ranked exact-retention and relative-refinement percentages over the 200 frozen GEANT2009 scenarios.

S3.3. Predeclared Scenario 0

The fixed illustrative scenario has source 29, $K = 32$, $H = 140$, ten eligible recipients, and observed recipients $\{0, 1, 2, 3, 5, 23, 30, 32\}$. All 34 candidates pass the deadline-insensitive static baseline and 20 remain under exact temporal feasibility. Fourteen candidates are rejected by shortest transit time. The true source remains retained and the exact stage makes 152 recipient-level calls.

Table S6. Candidate-level diagnostics for GEANT2009 scenario 0.

Candidate	True	$d_{\max}$	$\lambda_{\min}$	C_H	Calls	Outcome
0	no	98	400	8600	7	retained
1	no	110	400	6200	7	retained
2	no	56	400	18400	7	retained
3	no	90	400	13200	7	retained

Continued on next page

Table S6 continued

**Candidate**	**True**	$d_{\max}$	$\lambda_{\min}$	C_H	**Calls**	**Outcome**
4	no	80	400	16400	8	retained
5	no	99	400	12600	7	retained
6	no	120	400	4200	8	retained
7	no	139	400	400	8	retained
8	no	130	400	2200	8	retained
9	no	161	400	0	0	shortest transit time
10	no	197	400	0	0	shortest transit time
11	no	270	6	0	0	shortest transit time
12	no	274	50	0	0	shortest transit time
13	no	207	2	0	0	shortest transit time
14	no	236	100	0	0	shortest transit time
15	no	173	400	0	0	shortest transit time
16	no	130	400	2200	8	retained
17	no	119	400	5000	8	retained
18	no	238	250	0	0	shortest transit time
19	no	207	400	0	0	shortest transit time
20	no	172	400	0	0	shortest transit time
21	no	141	400	0	0	shortest transit time
22	no	129	400	2400	8	retained
23	no	112	400	5800	7	retained
24	no	120	400	4200	8	retained
25	no	160	100	0	0	shortest transit time
26	no	196	6	0	0	shortest transit time
27	no	163	200	0	0	shortest transit time
28	no	132	400	1800	8	retained
29	yes	140	400	200	8	retained
30	no	91	400	10000	7	retained
31	no	109	200	6400	8	retained
32	no	112	400	5800	7	retained
33	no	129	400	2400	8	retained

S4. Forty-Family Real-Network Transfer Study

The transfer study uses one latest eligible snapshot from each of 40 Internet Topology Zoo families represented by REPETITA benchmark graphs. The suite contains 3,392 nodes and 8,522 retained directed arcs. Graph sizes range from 50 to 197 nodes. Four self-loops in Interoute are removed because positive-transit self-loops are transport-inert. Non-self-loop parallel arcs in 18 networks are retained individually. All processed graphs are strongly connected. For each network independently, benchmark capacities and delays are mapped to controlled integer parameters by

$$c_e = \max\left\{1, \left\lfloor 32\frac{b_e}{b_{\max}} + \frac{1}{2}\right\rfloor\right\}, \qquad \tau_e = \max\left\{1, \left\lfloor 64\frac{d_e}{d_{\max}} + \frac{1}{2}\right\rfloor\right\}.$$

These are relative benchmark parameters, not claims of operator-measured absolute capacities or latencies. Before any source-detection results for the suite were computed, the policy froze five scenarios per network, master seed 20260903, $K = 256$, decode-and-replicate generation, the tenth non-source completion as H, eight positive-only recipients, all nodes as candidates, and descending shortest-transit-time recipient order for exact-stage early stopping.

Table S7. Primary equal-network-family results and prespecified capacity subgroups. For each network, the five scenarios are first averaged, after which the network-family means are weighted equally. Static retention, exact retention, relative refinement, and exact-stage fraction are reported as percentages. The exact-stage fraction is the fraction of candidate evaluations that reach the exact temporal oracle after preliminary screening. Confidence intervals bootstrap the 40 network rows.

Population	Networks	Static retention	Exact retention	Relative refinement	Exact-stage fraction	Calls per candidate
All families	40	99.27%	25.26%	74.45%	28.63%	2.03
Uniform	31	100.00%	17.18%	82.82%	22.31%	1.36
Heterogeneous	9	96.77%	53.09%	45.63%	72.32%	4.34

The 95% intervals for the equal-network mean are [98.39,99.93]% for static retention, [18.91,32.41]% for exact retention, and [67.45,80.95]% for refinement. All 200 scenarios retained their true source, and every exact retained set was a subset of the corresponding static set.

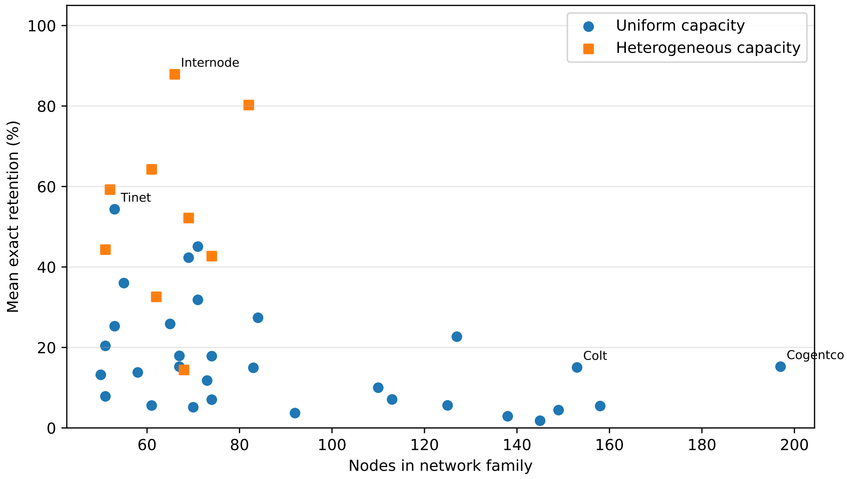

Figure S4. Mean exact retention versus network size for the 40 real-network topology families. Capacity class is descriptive and is not interpreted causally.

S4.1. Stage Decomposition and Computational Transfer

Table S8. Stage decomposition across all 16,960 candidate evaluations.

Outcome	Candidates	Fraction of all	Fraction of eliminated
Shortest-transit-time rejection	11,982	70.65%	90.03%
Static-cut rejection	53	0.31%	0.40%
Deadline-adjusted rejection	70	0.41%	0.53%
Exact temporal rejection	1,204	7.10%	9.05%
Retained	3,651	21.53%	not applicable

Most infeasible candidates are removed by the inexpensive shortest-transit-time screen, but exact temporal evaluation supplies an additional 1,204 rejections among the 4,855 candidates that reach the exact stage. The full suite makes 29,338 exact recipient calls, or 1.73 calls per candidate under candidate weighting and 2.03 under equal-network-family weighting.

S4.2. Per-Network Results

Table S9. Per-network results for the 40-family real-network study. Static retention, exact retention, relative refinement, and exact-stage fraction are means over five scenarios and are reported as percentages.

Network	n	Arcs	Cap.	Static retention	Exact retention	Relative refinement	Exact-stage fraction
AsnetAm	65	158	U	100.00%	25.85%	74.15%	25.85%
Bellsouth	51	132	U	100.00%	20.39%	79.61%	30.59%
RedBestel	84	202	U	100.00%	27.38%	72.62%	38.33%
BeyondTheNetwork	53	130	U	100.00%	25.28%	74.72%	35.85%
Cesnet201006	52	126	H	86.15%	59.23%	34.66%	81.54%
Cogentco	197	490	U	100.00%	15.23%	84.77%	23.35%
Colt	153	382	U	100.00%	15.03%	84.97%	23.66%
Columbus	70	170	U	100.00%	5.14%	94.86%	8.00%
Cudi	51	104	H	100.00%	44.31%	55.69%	75.29%
Dfn	58	174	U	100.00%	13.79%	86.21%	19.31%
DialtelecomCz	138	302	U	100.00%	2.90%	97.10%	3.48%
Esnet	68	184	H	97.06%	14.41%	85.15%	18.24%
Forthnet	62	124	H	100.00%	32.58%	67.42%	51.61%
Garr201201	61	178	H	93.77%	64.26%	32.18%	86.23%
Globenet	67	226	U	100.00%	15.22%	84.78%	22.39%
GtsCe	149	386	U	100.00%	4.43%	95.57%	5.91%
HiberniaGlobal	55	162	U	100.00%	36.00%	64.00%	49.09%
Intellifiber	73	194	U	100.00%	11.78%	88.22%	15.34%
Internode	66	156	H	100.00%	87.88%	12.12%	99.70%
Interoute	110	312	U	100.00%	10.00%	90.00%	15.45%
Ion	125	300	U	100.00%	5.60%	94.40%	8.48%
Iris	51	128	U	100.00%	7.84%	92.16%	8.63%
Deltacom	113	366	U	100.00%	7.08%	92.92%	7.79%
Latnet	69	148	U	100.00%	42.32%	57.68%	55.36%
Missouri	67	166	U	100.00%	17.91%	82.09%	24.18%

Continued on next page

Table S9 continued

**Network**	n	**Arcs**	**Cap.**	**Static retention**	**Exact retention**	**Relative refinement**	**Exact-stage fraction**
Oteglobe	83	204	U	100.00%	14.94%	85.06%	22.65%
Pern	127	258	U	100.00%	22.68%	77.32%	24.57%
Sinet	74	152	H	100.00%	42.70%	57.30%	71.62%
Surfnet	50	146	U	100.00%	13.20%	86.80%	16.40%
Switch	74	184	U	100.00%	17.84%	82.16%	23.51%
Syringa	74	148	U	100.00%	7.03%	92.97%	8.65%
TataNld	145	388	U	100.00%	1.79%	98.21%	2.62%
Telcove	71	140	U	100.00%	31.83%	68.17%	39.72%
Tinet	53	178	U	100.00%	54.34%	45.66%	55.85%
Tw	71	236	U	100.00%	45.07%	54.93%	57.75%
Ulaknet	82	164	H	100.00%	80.24%	19.76%	86.34%
Uninett2011	69	196	H	93.91%	52.17%	46.39%	80.29%
UsCarrier	158	378	U	100.00%	5.44%	94.56%	6.46%
UsSignal	61	158	U	100.00%	5.57%	94.43%	8.20%
VtlWavenet2011	92	192	U	100.00%	3.70%	96.30%	4.13%

S5. Delay-Normalization Fidelity in the Forty-Family Suite

The scale-64 delay mapping was audited independently of all source-identification results. Raw serialized delays were parsed as exact rational numbers. All-source Dijkstra computations compared native and reconstructed shortest distances for every ordered non-self node pair. For each pair, the audit also compared the set of all retained arc identities belonging to at least one shortest path. This preserves parallel arcs and tied shortest paths.

Table S10. Delay-normalization fidelity. The primary summary weights the 40 network rows equally. The pair-weighted summary pools 336,560 ordered pairs.

**Metric**	**Equal-network**	**Pair-weighted**
Mean absolute distance error	1.86%	1.64%
Median absolute distance error	1.05%	0.64%
Mean/pooled p95 error	3.41%	4.20%
Fraction within 5%	96.68%	96.02%
Fraction within 10%	98.95%	98.60%
Unchanged admissible arc set	91.15%	90.66%
Mean Jaccard similarity	96.65%	96.67%

The average behavior is stable, but individual outliers remain. Internode has mean all-pairs distance error 15.95%, although its median is 1.06%, p95 is 4.14%, and 96.41% of pairs remain within 5%. Its worst ordered pair has 6,185% distance error while retaining an identical admissible arc set. Tinet has the lowest unchanged-set fraction (40.28%) and mean Jaccard similarity (72.52%). These results motivate interpreting the suite as topology-transfer evidence under controlled benchmark parameters rather than deployment-level validation under original absolute latencies.

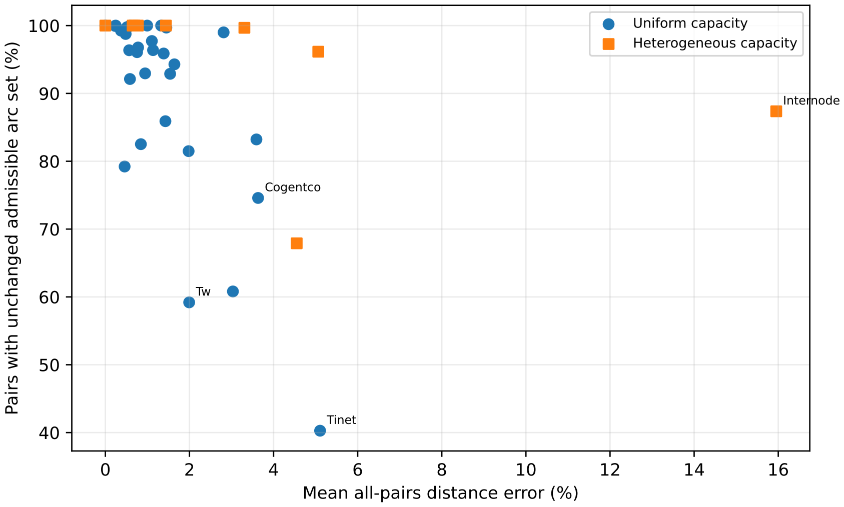

Figure S5. Per-network mean shortest-distance error and fraction of ordered pairs whose shortest-path admissible arc set is unchanged. Selected outliers are labeled.

Table S11. Per-network shortest-path fidelity. Error and p95 are all-pairs relative distance errors. Unchanged and Jaccard summarize admissible shortest-path arc sets.

Network	Mean error	p95	Within 5%	Unchanged	Mean Jaccard
AsnetAm	0.00%	0.00%	100.00%	100.00%	100.00%
Bellsouth	1.98%	3.33%	97.33%	81.49%	91.73%
RedBestel	3.59%	7.14%	83.33%	83.22%	96.85%
BeyondTheNetwork	1.54%	3.53%	97.39%	92.89%	98.17%
Cesnet201006	0.69%	1.80%	99.92%	100.00%	100.00%
Cogentco	3.63%	13.53%	80.42%	74.59%	88.86%
Colt	1.64%	3.99%	96.84%	94.29%	98.13%
Columbus	1.11%	1.37%	99.88%	97.72%	99.59%
Cudi	0.00%	0.00%	100.00%	100.00%	100.00%
Dfn	1.43%	2.77%	98.67%	85.90%	94.92%
DialtelecomCz	0.23%	0.58%	99.97%	99.95%	99.97%
Esnet	1.44%	1.28%	99.87%	100.00%	100.00%
Forthnet	0.77%	2.05%	99.05%	100.00%	100.00%
Garr201201	3.31%	4.24%	95.57%	99.67%	99.88%
Globenet	1.13%	1.83%	99.19%	96.38%	99.39%
GtsCe	0.78%	2.23%	99.30%	96.77%	98.78%
HiberniaGlobal	3.03%	8.82%	87.81%	60.81%	90.21%
Intellifiber	0.58%	1.55%	99.89%	92.12%	97.09%
Internode	15.95%	4.14%	96.41%	87.37%	94.83%
Interoute	0.84%	2.20%	99.20%	82.52%	95.76%
Ion	0.95%	2.08%	99.74%	92.95%	98.45%
Iris	0.52%	1.62%	100.00%	99.76%	99.88%
Deltacom	0.57%	1.54%	99.86%	96.37%	98.84%
Latnet	1.00%	3.25%	98.17%	100.00%	100.00%
Missouri	1.39%	3.34%	98.64%	95.88%	97.87%
Oteglobe	1.45%	3.89%	97.91%	99.71%	99.83%
Pern	1.33%	1.89%	100.00%	100.00%	100.00%
Sinet	5.06%	7.90%	86.93%	96.15%	98.46%
Surfnet	0.75%	2.24%	99.51%	96.08%	97.94%

Continued on next page

Table S11 continued

Network	Mean error	p95	Within 5%	Unchanged	Mean Jaccard
Switch	2.81%	3.91%	98.41%	99.00%	99.64%
Syringa	0.25%	0.76%	100.00%	100.00%	100.00%
TataNld	0.48%	1.37%	99.86%	98.76%	99.41%
Telcove	0.59%	1.86%	99.56%	100.00%	100.00%
Tinet	5.11%	15.87%	75.33%	40.28%	72.52%
Tw	1.99%	5.55%	93.16%	59.20%	80.53%
Ulaknet	0.65%	1.83%	99.85%	100.00%	100.00%
Uninett2011	4.55%	7.47%	90.88%	67.90%	83.04%
UsCarrier	0.46%	1.30%	99.80%	79.22%	95.94%
UsSignal	0.51%	1.48%	99.73%	99.62%	99.77%
VtlWavenet2011	0.37%	0.85%	99.98%	99.26%	99.66%

S6. RLNC Dynamics and Protocol-Feasibility Gap

This diagnostic explains the low refinement of the P3 RLNC regime and distinguishes exact coded temporal feasibility from two causal protocols. It reuses the original 100 frozen P3 base instances. They are 12-node directed graphs with $K = 4$, $H = 8$, capacities in $[1, 4]$, transit times in $[1, 3]$, and coding over $\mathbb{F}_{257}$. Every graph and candidate pair is simulated for 128 prespecified deterministic RLNC trials, giving 153,600 trials in total. The primary unit for intervals is the base instance rather than the candidate or trial.

S6.1. Static Saturation and RLNC Realization

Table S12. P3 static, exact, and causal-RLNC results in the full candidate domain. Here $\widehat{p}$ denotes the empirical RLNC success probability. The near-one mean success probability is reported as a probability rather than rounded to a percentage.

Quantity	Result
Candidate instances	1,200
Statically feasible candidates	1,200
Exact temporally feasible candidates	1,114
Shortest-transit-time rejections	24
Exact temporal rejections	62
Exact feasible with $\widehat{p} = 1$	1,087
Exact feasible with $0.95 \leq \widehat{p} < 1$	27
Exact feasible with $\widehat{p} < 0.95$	0
Mean empirical RLNC success probability among exact-feasible candidates	0.999691
Exact-infeasible successful RLNC trials	0

The static condition is saturated. All candidates pass it, with normalized static margin mean 6.35, median 7.00, and minimum 1.00. Exact coded temporal feasibility still retains 92.83% of candidates. Among the 1,114 exact-feasible candidates, every empirical success probability is at least 0.95, and 1,087 candidates succeed in all 128 trials. Thus weak refinement is explained by a permissive P3 operating regime and a broad exact coded-feasible region, not by widespread finite-field failure.

S6.2. Measured Dynamics and Decode-Before-Forward Counterfactual

Table S13. Causal RLNC dynamics and exact-to-decode-before-forward protocol gap.

Quantity	Result
Mean innovation fraction	10.54%
Mean partial-rank forwarding fraction	30.26%
Mean multi-predecessor recipient fraction	47.94%
Mean first-information-to-decode lag	1.13 slots
Decode-before-forward feasible among exact feasible	701/1,114
Exact feasible but decode-before-forward infeasible	413 (37.07%)
Exact-infeasible decode-before-forward successes	0

Approximately 30.26% of non-source relay transmissions occur while the relay has rank between one and $K-1$, and 47.94% of active recipients receive innovative packets from at least two predecessors. The exact-coded-to-RLNC operational gap is negligible under this frozen setting. The decode-before-forward restriction creates a substantial gap. A total of 413 of 1,114 exact-feasible candidates are infeasible under that counterfactual. The strongest descriptive associations with the decode-before-forward gap indicator are smaller exact margin ($\rho = -0.658$), greater partial-rank forwarding ($\rho = 0.553$), greater innovation fraction ($\rho = 0.545$), smaller outgoing capacity ($\rho = -0.509$), and tighter deadline slack ($\rho = -0.492$). These associations are descriptive and do not establish causality.

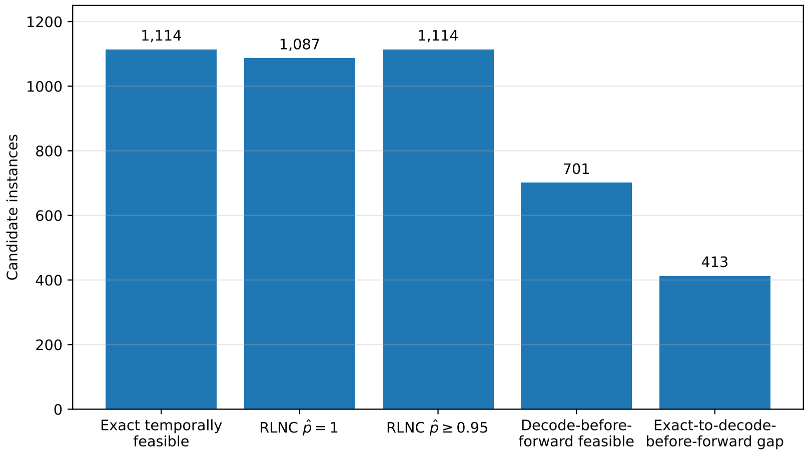

Figure S6. Exact temporal feasibility, empirical RLNC realization, and decode-before-forward feasibility in the P3 diagnostic. The exact-to-decode-before-forward gap is a subset of the exact-feasible population and not an additional outcome category.

S6.3. Fixed Pipeline Witness and Bootstrap Estimates

On the predeclared two-hop chain $s \to v \to a$ with unit capacities and transit times, $K = 2$, and $H = 3$, causal partial-information forwarding sends the first symbol onward at time 1 and the second at time 2, so a decodes at time 3. The exact temporal capacity is two. Decode-before-forward waits until v has decoded both symbols and completes at a only at time 4. This witness isolates the pipeline mechanism without selecting a favorable case after observing the population results.

Table S14. Primary estimates obtained by weighting the 100 frozen P3 base graphs equally. Intervals use 10,000 deterministic bootstrap repetitions over the 100 base-graph rows. Empirical fractions are reported as percentages, except for the near-one RLNC success probability, which is retained to six decimal places.

Metric	Estimate	95% interval
Mean exact retention	92.83%	[90.50,94.92]%
Mean RLNC success probability among exact feasible	0.999646	[0.999436,0.999809]
Mean exact-to-decode-before-forward gap	37.86%	[33.58,42.32]%
Mean partial-rank forwarding fraction	30.26%	[28.99,31.58]%

The original P3 generation loop required at least four decoded non-source recipients. Exact replay showed 100 attempts, 100 acceptances, and no rejected graph, so this condition introduced no rejection-conditioning distortion in the frozen population.

References

1. Knight, S.; Nguyen, H.X.; Falkner, N.; Bowden, R.; Roughan, M. The Internet Topology Zoo. *IEEE J. Sel. Areas Commun.* **2011**, *29*, 1765–1775.
2. Gay, S.; Schaus, P.; Vissicchio, S. REPETITA: Repeatable Experiments for Performance Evaluation of Traffic-Engineering Algorithms. *arXiv* **2017**, arXiv:1710.08665.

Disclaimer/Publisher's Note: The statements, opinions and data contained in all publications are solely those of the individual author(s) and contributor(s) and not of MDPI and/or the editor(s). MDPI and/or the editor(s) disclaim responsibility for any injury to people or property resulting from any ideas, methods, instructions or products referred to in the content.
